# Target Binding of Black Phosphorus Nanomaterial to Polo‐Like Kinase 1 for Cancer Chemotherapy: A Mutual Selection of Nanomaterial and Protein

**DOI:** 10.1002/EXP.20240143

**Published:** 2025-09-26

**Authors:** Fangfang Liu, Zhong‐Da Li, Yanqiao Zeng, Xiaofeng Wang, Yingnan Liu, Qi Li, Wenhe Luo, Xiaoman Suo, Yaqing Xu, Feng Yuan, Dan Zhang, Wuqiong Zhang, Shengyong Geng, Xue‐Feng Yu, Guofang Zhang, Yang Li

**Affiliations:** ^1^ Laboratory of Inflammation and Vaccines Shenzhen Institutes of Advanced Technology Chinese Academy of Sciences Shenzhen China; ^2^ Laboratory of Immunology and Nanomedicine & China‐Italy Joint Laboratory of Pharmacobiotechnology for Medical Immunomodulation Shenzhen Institutes of Advanced Technology Chinese Academy of Sciences Shenzhen China; ^3^ Department of Biosciences and Medical Biology Division of Allergy and Immunology Paris Lodron University of Salzburg Salzburg Austria; ^4^ Universitat Autònoma de Barcelona (UAB) Barcelona Spain; ^5^ Materials and Interfaces Center Shenzhen Institutes of Advanced Technology Chinese Academy of Sciences Shenzhen China; ^6^ Key Laboratory of Biomedical Imaging Science and System Chinese Academy of Sciences Shenzhen China

**Keywords:** black phosphorus nanomaterials, PLK family, PLK1 inhibitor, protein structure, targeted interaction of nanomaterial with protein

## Abstract

The intrinsic properties of black phosphorus (BP) nanomaterials (NMs) enable them for targeted binding to polo‐like kinase 1 (PLK1), thus inhibiting its kinase activity. However, the mechanism and targeted binding sites underlying this interaction remain unclear. To elucidate the critical properties of PLK1 that facilitate its interaction with BPNMs, the binding ability of BPNMs was compared across PLK family proteins. Although BPNMs exhibited a weak binding affinity for PLK3, PLK1 demonstrated the most favorable physicochemical properties for its binding. Factors as surface charge, hydrophobicity, secondary and three‐dimensional structures significantly affected the interaction of PLK family proteins to BPNMs. The binding affinity was primarily determined by amino acid residues at the binding interface, where arginine and proline played critical roles in mediating the interaction of BPNMs‐PLK1. BPNMs inhibited PLK1 activation by binding to key residues of the kinase domain, including S49, Y203, D204, E206, and R207. In conclusion, this study elucidates the molecular basis of the specific interaction between BPNMs and PLK1, highlighting the pivotal role of the amino acid residues in NM‐protein binding. This work demonstrates that NM‐protein interactions are a mutual selection and driven by the physicochemical properties of both proteins and NMs.

## Graphical abstract

This study investigates the effect of protein properties on the nanomaterial‐protein interaction and elucidates the molecular mechanisms underlying the targeted inhibition of polo‐like kinase (PLK1) by black phosphorus nanomaterials (BPNMs). The specific targeting inhibition is attributed to the intrinsic properties of both the PLK1 protein and the BPNMs. Notably, BPNMs specifically bind to PLK1, while exhibiting no binding affinity to homologous proteins within PLK family. BPNMs effectively suppress PLK1 activity by binding to critical amino acid residues in both the kinase domain and polo‐box domain. Specifically, BPNMs target to the residues S49, known as a phosphorylation site, as well as to the residues Y203, D204, E206, and R207, which are all located within the activation loop of the kinase domain and critical for PLK1 activation. Additionally, R337 is situated in the D‐box region, playing a vital role in PLK1 degradation, and is also specifically attached to BPNMs. By specifically targeting PLK1, BPNMs disrupt the normal cell cycle and present themselves as a potential candidate for targeted chemotherapeutic agents.

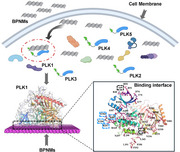

## 1 Introduction

Nanomedicines showed unique advantages in clinical efficacy compared with traditional small molecule drugs, but only a limited number of nanomedicines have been commercialized globally [1]. The main hurdle in moving nanomedicines from the bench to clinical applications is the limited knowledge of their fundamental biological mechanisms [2]. Due to the small size and large surface area, NMs can absorb many biomolecules after entering biological fluids, resulting in new identification of the biomolecule coated NMs in bodies [3]. Biomolecule layers coated spontaneously on the surface of NMs were referred to as biocorona or protein corona [4]. Protein corona alters the surface of NMs, affecting their pharmacological and toxicological effects as well as their therapeutic or diagnostic potentials [5]. Protein corona can be roughly divided into “soft corona”, in which rapid dynamic exchange of biomolecules between the medium and the particles predominates, as well as “hard corona”, in which the biomolecules bind with high affinity to the particle surface [6]. The composition and characteristics of the protein corona varied from different NMs due to their chemical composition and physicochemical properties, as well as the protein composition of the biological environment [7], suggesting that the formed protein corona may depend on both the NMs and the proteins.

The physicochemical properties of NMs play an important role in the NM‐protein interaction, including the chemical composition, size, surface charge, and hydrophobicity of NMs [3, 8]. Citrate‐coated silver NMs and chitosan NMs with small size exhibited stronger interactions with human serum albumin than the NMs with large size [9], while there is no significant difference in the interaction of soluble yeast protein extracts with silica NMs of different sizes [10]. Therefore, there are no consistent results regarding the size effect on the NM‐protein interaction. Alternatively, physicochemical properties of proteins (e.g., the surface charge) also significantly affect their binding to NMs. It has been proven that the secondary structure of protein is critical in defining the NM protein interface with mesoporous silica NMs [8a]. To be noted, the interaction with NMs could also induce the conformational change of proteins, which leads to inactive proteins or the disruption of protein–protein interactions [11]. Thus, the NM‐protein interactions provide a possibility that the NMs could be used as a protein functional inhibitor.

In recent years, it has been reported that the intrinsic property of NMs endows them to specifically bind with drug target proteins, significantly increasing the clinical translational potential of these inorganic NMs [12]. During the pandemic period of COVID‐19, the antiviral ability of NMs against severe acute respiratory syndrome coronavirus 2 (SARS‐CoV‐2) has been widely studied. The ultrathin two‐dimensional (2D) CuInP_2_S_6_ nanosheets that selectively bind to the spike protein receptor‐binding domain (RBD) of SARS‐CoV‐2 [13]. Additionally, the 3 nm cerium oxide NMs (CeO_2_@3) insert the 5 nm spike protein trimer cavity and tightly bind with the RBD [14]. Meanwhile, the flat 𝜶‐cobalt hydroxide nanosheets bind with RBD due to their higher proportion of trivalent cobalt [15]. These properties endow these NMs to inhibit viral infection in ACE2‐expressing cells by blocking the RBD‐ACE2 interaction. Notably, the binding sites of NM‐protein are far more diverse than those of small molecule drugs, making antiviral NMs more promising candidates for broad‐spectrum therapies against viral variants. This suggests that NMs can interact with a broader range of active sites on target proteins compared to small molecule drugs, potentially enhancing their efficacy in targeting diverse viral strains.

Polo‐like kinase 1 (PLK1) is a member of PLK family, which includes the conserved Ser/Thr protein kinase PLK1‐5. These proteins play a crucial role in regulating the cell cycle and share a similar structure [16]. PLK1 is an attractive target for cancer therapy because it is overexpressed in most human cancers. An elevated PLK1 leads to multiple defects in mitosis and cytokinesis, impairment of supernumerary centrosomes and cell cycle checkpoints, which in turn induces aneuploidy and tumorigenesis [16, 17]. In reverse, the inhibition of PLK1 could cause mitotic blockage and apoptosis in most cancers [17b]. PLK1 consists of an amino‐terminal catalytic kinase domain (KD) and a carboxy‐terminal polo‐box domain (PD) of two polo boxes, which is typical for the PLK family [16]. The specificity of PLK1‐dependent biochemical reactions is regulated by two sequential steps, including PD‐dependent substrate binding and KD‐dependent substrate phosphorylation; therefore, suppression of either of the two domains is sufficient to inhibit the PLK1 function in vivo [18]. Currently, some small‐molecule and peptide‐derived inhibitors of PLK1 have been developed, but they have only achieved limited efficacy [19]. KD inhibitors often exhibit non‐specificity by binding to other proteins that share a similar kinase domain, leading to dose‐dependent toxicity. In contrast, PD inhibitors demonstrate high specificity but suffer from poor membrane permeability and low bioavailability [20]. Ideally, an effective PLK1 inhibitor should be capable of binding to both the KD and PD with good bioactivity [21]. Our previous study demonstrated that black phosphorus nanomaterials (BPNMs) present a novel approach to cancer chemotherapy by inducing significant G2/M phase arrest in tumor cells [22]. We further investigated the underlying mechanisms through which BPNMs exert their chemotherapeutic effects and discovered that they can directly target PLK1 [23]. BPNMs can induce aggregation of PLK1 in the cytoplasm, reducing its cytosolic mobility and preventing its recruitment to centrosomes. The deactivation of PLK1 compromises centrosome integrity, leading to centrosome fragmentation and formation of multipolar spindles in mitosis. These abnormalities induce a significant G2/M phase arrest in tumor cells, ultimately leading to apoptosis [23]. In short, BPNMs have been proven as an effective PLK1 inhibitor for anti‐tumor treatment both *in* *vitro* and *in* *vivo*. Importantly, this study is the first attempt to propose a nano‐inhibitor of PLK1, thus leading to a remarkably promising direction for future cancer chemotherapy.

However, the specificity of the interaction between BPNMs and PLK1 remains to be elucidated, as well as the precise molecular interface mediating the BPNM‐PLK1 binding. The specificity and selectivity of the binding between targeted proteins and inhibitors are crucial for understanding the underlying molecular mechanisms and paving the way for potential clinical applications. Therefore, addressing these key questions is essential for advancing the translational potential of BPNMs as a promising chemo‐drug candidate targeting PLK1. In addition, a fast rate of BP degradation also limits their efficiency for cancer chemotherapy [24]. Besides instability, the bioactivity of BP (e.g., target efficiency) is also compromised by surface oxidation [25]. To address these concerns in the further development of the nano‐drug candidate, for example, low biocompatibility, poor tumor targeting, and instability, strategies have been developed by employing BPNMs in combination with various materials, significantly enhancing its anti‐tumor efficacy as a photosensitizer or an autophagy inhibitor [26]. In addition, the cell membrane‐coated NMs (CNMs) were proposed and demonstrated as a powerful approach in recent years [27]. Myeloid‐derived cells were considered as a promising approach for selective delivery of cancer therapeutics due to their chemotic properties, which enable myeloid cells to effectively navigate and respond to the tumor microenvironment (TME) [28]. Therefore, the combination of the CNMs approach with the tumor‐targeting capacity of myeloid‐derived cells presents a promising solution to enhance the chemotherapy efficiency of BPNMs.

**SCHEME 1:**
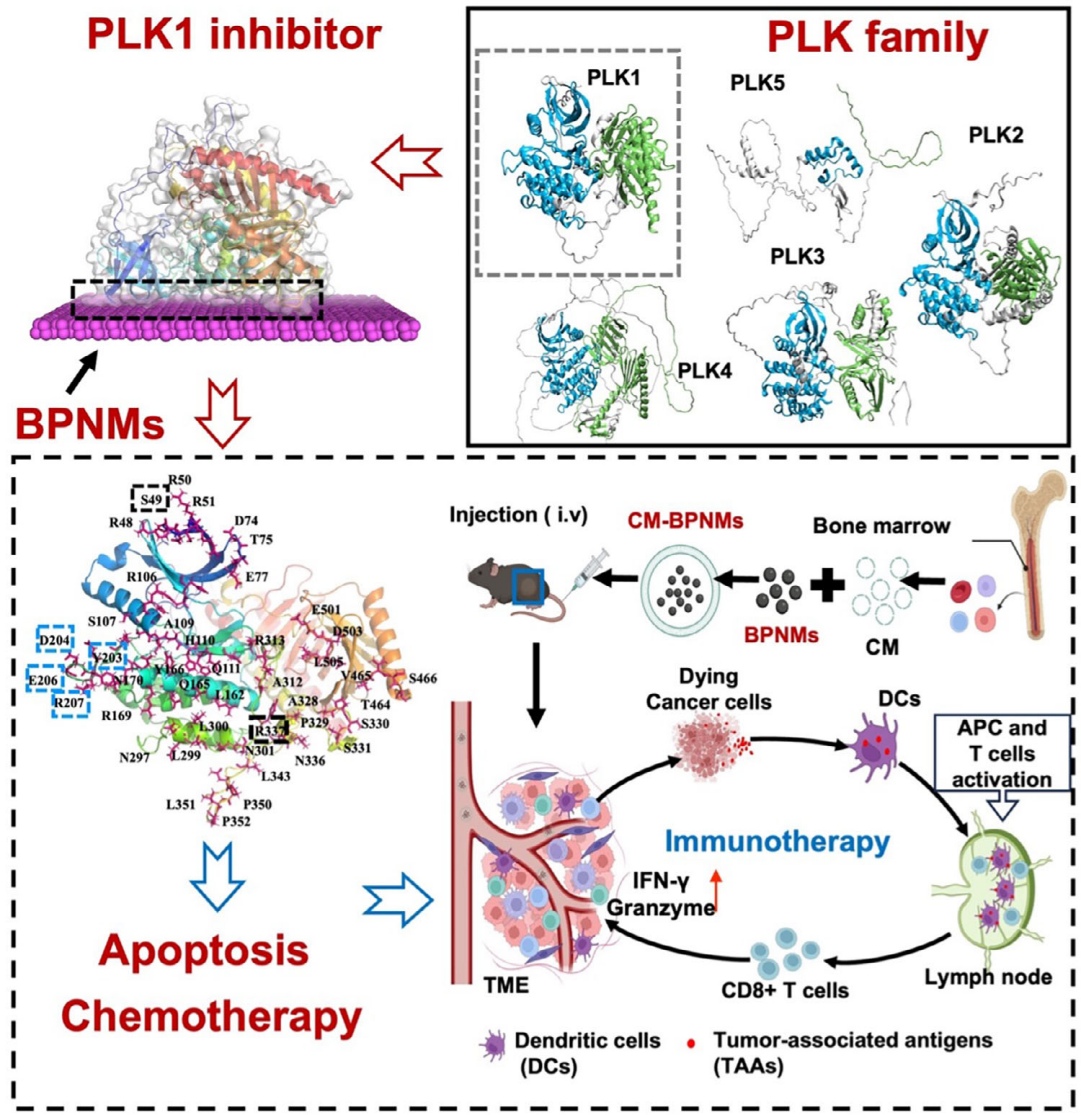
Schematic illustration of the mechanisms of BPNMs as the specific inhibitor of PLK1 (a key kinase in mitosis) for cancer chemotherapy. BPNMs bind with PLK1 by their two functional domains, making them a promising inhibitor. Specifically, BPNM binds to several critical amino acids of PLK1. These include S49, a phosphorylation site, as well as the residues Y203, D204, E206, and R207 within the activation loop that are critical for PLK1 activation. Additionally, R337 located in the D‐box region, plays an important role in PLK1 degradation. By targeting these critical residues, BPNMs effectively disrupt the normal cell cycle. Furthermore, cell membranes‐coated BPNMs (CM‐BPNMs) derived from myeloid cells demonstarted enhanced chemotherapeutic efficiency in vivo, functioning as the PLK1 inhibitor and concurrently providing immunotherapeutic benefits by activation of dendritic cells in melanoma‐bearing mouse after intravenous injection.

This study aimed to investigate the specificity of BPNM binding to PLK1 and elucidate the factors (e.g., the physicochemical properties of both NMs and proteins) affecting the BPNM‐PLK1 interaction. Additionally, the study sought to evaluate the efficiency of the myeloid‐derived cell membranes coated BPNMs (e.g., CM‐BPQDs) in cancer chemotherapy. The data showed that BPNMs specifically bind to PLK1 within the PLK family in a size‐independent manner (Scheme 1), demonstrating a mutually selective interaction driven by both the proteins and the NMs. The binding capacity of PLK proteins to BPNM was influenced by multiple key factors. These included surface charge, grand average of hydropathicity, 3D and secondary structure, and protein localization. Among the PLK family members, the physicochemical properties of PLK1 were optimal for its interaction with BPNMs, indicating that the PLK1 was the preferred binding target of BPNMs. In detail, the molecular dynamics (MD) simulation data showed that BPNMs bound to S49, which acts as a phosphorylation site, as well as to the residues Y203, D204, E206, and R207 located within the activation loop of the kinase domain, which is critical for PLK1 activation. Additionally, R337 plays an important role in PLK1 degradation (Scheme 1). Meanwhile, the myeloid‐derived cell membranes (CM) coating addresses the limitations of pristine BPNMs for a systematic administration for cancer treatment. This study further establishes the theoretical foundation of the specific NM‐protein interaction, uncovers the molecular basis of specific targeting of PLK1 by BPNMs, and enhances their potential as specific PLK1 inhibitors in cancer chemotherapy.

## 2 Materials and Methods

### 2.1 Cell Lines and Animals

The human embryonic kidney 293 (293T) cells, murine melanoma cell line B16F10, and human epidermal keratinocytes (HEKa) cells were obtained from American Type Culture Collection (ATCC). B16F10 cells were cultured with Roswell Park Memorial Institute medium 1640, 293T and HEKa cells were cultured in DMEM medium with 10% fetal bovine serum and 1% penicillin/streptomycin at 37°C in 5% CO_2_. The 6‐week‐old female C57BL/6 mice were obtained from Guangdong Vital River Laboratories (Guangdong, China). All animal experiments were performed under the Guide for Care and Use of Laboratory Animals (approval ID SIAT‐IACUC‐20220329‐YYS‐LY‐A2134 from the Institutional Animal Care and Use Committee (IACUC) of Shenzhen Institutes of Advanced Technology (SIAT), CAS).

### 2.2 Synthesis of BPNMs

The BPNS and BPQD were synthesized using a liquid exfoliation method as previously described [29]. Briefly, 25 mg of BP powder (gif from MoPhos) was mixed with 25 mL of *N*‐methyl pyrrolidinone (NMP) (Aladdin Reagents), and ultrasonicated at 19–25 kHz for 1.5 h (BPNS) or 4 h (BPQD) with 1200 W. Then, the oversized particles were removed by centrifugation at 2000×*g* for 10 min. Furtherly, the supernatant was sonicated for 8 h (BPNS) or 16 h (BPQD) at 300 W in the ice bath. Finally, the supernatant containing BPNS (100–300 nm) or BPQD was collected after centrifugation at 7500×*g* for 10 min. The BPNS and BPQD were isolated by centrifugation at 21,000×*g* for 10 min before the next experiments. The precipitate was rinsed twice with 100% ethanol and water, respectively.

### 2.3 Characterizations of BPNMs

The morphologies of BPNS and BPQD were observed by transmission electron microscope (TEM) (FEI Talos F200x, USA). The size of BPNMs was calculated by measuring 50 particles randomly under TEM. The zeta potential of BPNMs was detected by dynamic light scattering (DLS) on Zetasizer Nano ZS90 (Malvern, UK). The thickness of BPNS was determined by atomic force microscopy (AFM) (RTESP‐300, BRUKER, USA). The stability of BPQD was evaluated with alterations of concentrations in water for 7 days. The concentration of BPNS and BPQD was calculated as following equations according to the absorbance (OD) at 808 nm by (Multiskan GO, Thermo Fisher Scientific, Inc.).

$$\text{Concentration}\left(\text{BPNS,}\;\mu \text{g/mL}\right)=\text{OD}/14.8\times 1000$$

$$\text{Concentration}\left(\text{BPQDs,}\;\mu \text{g/mL}\right)=\text{OD}/18.0\times 1000$$

### 2.4 Characterizations of Proteins in PLK Family

The physicochemical parameters were computed using Expasy ProtParam according to their sequence of proteins in PLK family including theoretical pI, instability index, aliphatic index, and grand average of hydropathy (GRAVY). The secondary and three‐dimensional (3D) structures were obtained from AlphaFold Protein Structure Database based on their Uniprot ID (PLK1, P53350; PLK2, Q9NYY3; PLK3, Q9H4B4; PLK4, J3KR84; PLK5, A0A286YFL1).

### 2.5 Plasmid Construction

To facilitate the construction of plasmids encoding the Polo‐like kinase (PLK) family proteins, we utilized the following UniProt accession numbers to obtain their corresponding protein sequences: PLK1 (P53350), PLK2 (Q9NYY3), PLK3 (Q9H4B4), PLK4 (J3KR84), PLK5 (A0A286YFL1). For the generation of plasmids pET‐28a (+)‐PLK1‐full length (FL), kinase domain (KD) and polo box domain (PD), the coding sequence of PLK1‐FL, PLK1‐KD, and PLK1‐PD was inserted into the pET‐28a (+) vectors with His tag by *Eco*RI (Thermo Fisher Scientific, Inc.), respectively. The coding sequence of FL, KD and PD of PLK1, PLK2, PLK3, PLK4 and PLK5 were inserted into the pCMV‐Tag 2B vectors with Flag tag to construct the plasmids of pCMV‐Tag 2B ‐PLK1‐FL, ‐PLK1‐KD and ‐PLK1‐PD, ‐PLK2‐FL, ‐PLK2‐KD and ‐PLK2‐PD, ‐PLK3‐FL, ‐PLK3‐KD and ‐PLK3‐PD, ‐PLK4‐FL, ‐PLK4‐KD and ‐PLK4‐PD, as well as ‐PLK5‐FL, ‐PLK5‐KD and ‐PLK5‐PD.

### 2.6 Pull down Assay

Lipomaster 2000 Transfection Reagents (Vazyme, China) was used to transfect the constructed plasmids with Flag tag including pCMV‐Tag 2B‐PLK1‐FL, ‐PLK1‐KD and ‐PLK1‐PD, ‐PLK2‐FL, ‐PLK2‐KD and ‐PLK2‐PD, ‐PLK3‐FL, ‐PLK3‐KD and ‐PLK3‐PD, ‐PLK4‐FL, ‐PLK4‐KD and ‐PLK4‐PD, as well as ‐PLK5‐FL, ‐PLK5‐KD and ‐PLK5‐PD in 293T cells for 24 h for protein expression. Then, the cells were lysed with lysis buffer (Yeasen, China) containing a protease inhibitor cocktail (Roche, Switzerland) for 30 min and centrifuged 12,000 rpm for 5 min at 4°C to collect the proteins in the supernatant. The concentrations of proteins were determined with the Enhanced BCA Protein Assay Kit (Beyotime, China) for the next analysis.

In total, 300 µg proteins were incubated with 10 µg BPNMs in 1 mL volume for 2 h and then centrifugated at 6000 rpm at 4°C for 5 min to obtain precipitates. After washing three times, the obtained precipitates were boiled with loading buffer at 100°C for 10 min for western blot to assay the binding of BPNS and BPQDs with the protein of PLK1‐FL, ‐PLK1‐KD and ‐PLK1‐PD, ‐PLK2‐FL, ‐PLK2‐KD and ‐PLK2‐PD, ‐PLK3‐FL, ‐PLK3‐KD and ‐PLK3‐PD, ‐PLK4‐FL, ‐PLK4‐KD and ‐PLK4‐PD, as well as ‐PLK5‐FL, ‐PLK5‐KD and ‐PLK5‐PD. In brief, the proteins were separated with 10% SDS‐PAGE and transferred to a PVDF membrane (Millipore, USA). The specific protein was detected with anti‐Flag (Proteintech, China) and analyzed with an Amersham Imager AI600 (GE, USA).

### 2.7 Protein Purification

The pET‐28a (+)‐PLK1‐FL, PLK1‐KD, and PLK1‐PD plasmids were transformed into *Escherichia coli* BL21(DE3) cells (Yeasen, China) for protein expression. The BL21(DE3) cells with the plasmids were incubated in LB medium with kanamycin at 37°C with shaking until the OD_600_ reached 0.6–0.8. The IPTG (1 mM for PLK1‐FL and PLK1‐PD, 0.1 mM for PLK1‐KD) was added into the LB medium to induce the expression of proteins. The cells were further grown for 6 h (PLK1‐FL and PLK1‐PD) at 37°C and 16 h (PLK1‐KD) at room temperature after induction. Then, the cells were harvested by centrifugation for 10 min at 10,000 rpm (Multifuge X1 pro, Thermo Fisher, USA) at 4°C. Protein purification was performed for the cell pellets according to the protocol of HisSep Ni‐NTA Agarose Resin (Yeasen, China). Finally, SDS‐PAGE was used to quantify the yield of protein expression and purification (Comassie staining). The purified protein was stored at ‐80°C for the next analysis.

### 2.8 Biolayer Interferometry (BLI) Analysis

The Biolayer Interferometry (BLI) technique was used to measure the binding affinity of BPNS or BPQDs to PLK1‐FL, PLK1‐KD, and PLK1‐PD, using an Octet RED96e system (FortéBio, Bohemia, NY, USA). PBST (0.01% Tween 20 in PBS) was used as the binding buffer. First, a baseline was established for 180 s. PLK1‐FL, ‐KD, and ‐PD with His‐tag were immobilized on the surface of His1K sensor chip (immobilization time 300 s). Then, sensors were immersed into the BPNS and BPQDs, and their binding was assessed with an association time of 600 s and a dissociation time of 600 s. The concentrations of proteins were 10 µg mL^−1^, and those of BPNS and BPQDs were 500, 250, 125, 62.5, 31.25, and 15.63 nM, respectively. The data were analyzed with the Data Analysis 11.0 Software. The parameters of binding affinity were calculated by fitting the curves using the 1:1 kinetic binding model, including affinity constants (*K*_d_), association rate constants (*K*_on_), and dissociation rate constants (*K*_off_).

PLK1 (25 µg mL^−1^) was modified with biotin firstly and immobilized on the surface of SSA sensor chip (immobilization time 600 s) to assess the effect of BPNS on the binding of PLK1 and its substrate (ATP). Then, sensors were immersed into the BPNS (25 µg mL^−1^) to bind with PLK1 (immobilization time 300 s). Finally, the sensors were moved to wells containing 8 mM ATP for evaluating the effect of BPNS on the function of PLK1. The data were analyzed with the Data Analysis 11.0 Software.

### 2.9 Molecular Dynamic (MD) Simulation System

The BPNS (30 nm) were assembled using CCDC Mercury2022.2.0 software [30]. The PLK1 (Uniprot ID: P53350) and PLK3 (Uniprot ID: Q9H4B4) were predicted using Alphafold2. Our previous research demonstrated that BPNS interacts with PLK1 during the G2 phase, leading to a reduction in its activity and suggesting that PLK1 predominantly adopts a partially inhibited conformation. We further modeled this partially inhibited conformation of PLK1 in our MD simulations, highlighting the interaction between R136 in the kinase domain and P403 in the polo‐box domain. The two functional domains, PLK1‐KD and PD, were obtained from the PDB ID database as 3FC2 and 1Q4K, respectively. The water molecules and extraneous heteroatoms were removed from PLK1 protein to retain the structure of the protein using UCSF Chimera [31]. The force field used to describe the protein was AMBER14SB [32]. Amino acid PK values were calculated and assigned under neutral conditions (PH 7.0) using the H++3 online tool [33]. The initial structure of the protein and BPNS complex was constructed, with the Packmol atomic tolerance 2.0 Å using the PackMOL modeling tool [34]. Proteins are randomly oriented vertically above the BPNS. At last, the TI3P water model and Na^+^ and Cl^−^ were added to neutralize the system and maintain a physiological concentration of 0.15 M [35].

Molecular dynamics simulations were performed for 100 ns to investigate the effect of BPNS on the protein structure using the Gromacs 5.1.5 open‐source software package [36]. The system was established under well‐defined conditions: 289.15 K (25°C), pH 7, and 1 bar pressure. Production simulations were conducted for 100 ns with a 2‐fs time step. All simulations were visualized using PyMOL2.04 software [37]. The GMX module was used to calculate the radius of gyration, as well as the root mean square deviation (RMSD) and root mean square fluctuation.

### 2.10 Secondary Structure Characterization of PLK1 by Circular Dichroism (CD)

The secondary structure of the PLK1 detected in the absence or presence of BPNS was assessed by CD spectrum (J‐810, JASCO). PLK1 (200 µg mL^−1^) was incubated with BPNS (1, 10, and 100 µg mL^−1^) for 2 h to perform the CD assay using a cuvette with a thickness of 1 mm. CD spectra were collected between 190 and 250 nm. The spectral data were processed using the CD Tool software (http://cdtools.cryst.bbk.ac.uk) to subtract the baseline (between 235 and 250 nm). Finally, normalized data were analyzed using BeStSel to calculate the percentage of secondary structure, and the smoothed data are shown.

### 2.11 Apoptosis Assay in vitro

B16F10 cells were cultured in 12‐well plates with a density of 5 × 10^4^ cells per well and treated with 10, 20, and 40 µg mL^−1^ BPQDs for 24 h. Cells and supernatant were collected and labeled with PI and Annexin V‐FITC following the protocol of Annexin V‐FITC/PI Cell Apoptosis Detection Kit (Yeasen, China). Then, cells were analyzed by flow cytometry (CytoFLEX with, Beckman Coulter).

### 2.12 Extraction of Cell Membranes

To isolate cell membranes, the myeloid‐derived cells were obtained from excised tibiae and femurs of C57BL/6J mice. The cells were filtered with 70 µm cell strainer, then centrifugated at 1000 rpm for 5 min to remove supernatant. The cells were resuspended with 1 mL lysis buffer (Yeasen, China) for 15 min to break the cells in an ice bath. The cells were then freeze‐thawed three times with liquid nitrogen and centrifugated at 700 rpm for 10 min to collect supernatant. Then the supernatant was centrifugated at 13,500 rpm for 30 min. Finally, the precipitates were kept and resuspended with 1 mL PBS to detect the concentrations of cell membranes with the Enhanced BCA Protein Assay Kit (Beyotime, China) for the next step.

### 2.13 CM‐BPQDS Extrusion and Characterization

The CM‐BPQDs were assembled by direct extrusion of cell membranes from the myeloid‐derived cells coated with BPQDs. Briefly, BPQDs and cell membrane vesicles were mixed in a 2:1 ratio and sonicated for 10 min, then physically extruded 11 times using a mini extruder with a polycarbonate 400 nm porous membrane (Avestin, Canada) to obtain CM‐BPQDs.

The size and zeta potential of the cell membrane and CM‐BPQDs were assayed by DLS on Zetasizer Nano ZS90 (Malvern, UK). The morphology of CM‐BPQDs was obtained under transmission electron microscopy (TEM) (HITACHI HT7800, Japan), after staining with 1% phosphotungstic acid solution (w/v; Rhawn, China). The stability of BPQDs and CM‐BPQDs was evaluated over 7 days by monitoring their change of UV–vis absorption spectra.

The chemokine receptors such as C‐C chemokine receptor type 2 (CCR2) and C‐X‐C chemokine receptor type 4 (CXCR4) on myeloid cells were crucial for promoting cell migration and retention in the tumor microenvironment [38]. Therefore, CRR2 and CXCR4 were detected on CM‐BPQDs by flow cytometry with PE‐anti‐mouse CD192 (CCR2) and APC‐anti‐mouse CD184 (CXCR) antibodies (BioLegend, USA) to evaluate their targeting potential.

### 2.14 Cell Viability Assay

Human epidermal keratinocytes (HEKa) were planted in 96‐well plates with 8 × 10^3^ cells/well density, respectively. After overnight incubation, cells were exposed with 5, 10 and 20 µg mL^−1^ CM‐BPQDs for 24 h. Then cells were incubated with fresh DMEM with 10% CCK8 solution which can be transformed in formazan by enzymes of living cells for 1‐4 h (until OD value around 1.0) at 37°C. The absorbance was measured at 450 nm using a microplate reader (Multiskan GO, Thermo Fisher Scientific, Inc.). The cell viability was normalized with the control group.

### 2.15 The Anti‐Tumor Experiment *in* *v* *ivo*

About 1 × 10^5^ B16F10 cells were injected subcutaneously into the flank of female C57BL/6J mice (6–8 weeks old) to construct melanoma bearing mouse model. In our previous study, BPNMs were administrated to mice in situ (25 µg). In the present study, CM‐BPQDs were administrated to mice by intravenous injection (i.v.). The two to five times dose of intertumoral injection (i.t.) was used for i.v. [39]. The mice were divided randomly into three groups to investigate the anti‐tumor effect of CM‐BPQDs. The treatments are as follows: PBS (i.v.), BPQDs (25 µg, i.t.), and CM‐BPQDs (100 µg, i.v.). Mice with 50–150 mm^3^ melanoma were administrated with different treatments every other day for four times. The length (*L*), width (*W*), and height (*H*) of tumors were recorded using digital calipers every 2 days to calculate the tumor volume as *L* × *W* × *H* × 0.52. Mice were sacrificed before the tumor volume reached 2000 mm^3^ to collect tumor for further analysis.

### 2.16 Histology and TUNEL Assay

Tumor tissues were fixed with 4% polyoxymethylene for 48 h, then immersed in 75% (v/v) alcohol for 4 h, 85% and 90% alcohols for 2 h separately, 95% alcohol for 1 h, and 100% alcohol for 30 min twice to dehydrate. To degrease, samples were incubated in benzyl alcohol for 10 min, then in xylene for 10 min twice, finally in 65°C melting paraffin for 1 h three times. The wax‐soaked tissue is embedded in the embedding machine and solidified at −20°C. The blocks were cut into 4 µm slices, then put into the glass slides and dried at 60°C for the next step.

The slides were stained with DAPI and terminal deoxynucleotidyl transferase (FITC‐12‐dUTP) according to the protocol of Fluorescein (FITC) TUNEL Cell Apoptosis Detection Kit (Servicebio, China). The images were acquired with the stimulated emission depletion microscope under 20× objectives (STEDYCON Abberior Instruments GmbH, Göttingen, Germany).

### 2.17 Immune Response and Remodulation of Immunosuppressive TME by CM‐BPQDs

Tumors were collected to investigate different infiltration immune cells. First, tumors were digested with cell culture media containing 0.5 mg mL^−1^ type IV collagenase, 0.1 mg mL^−1^ hyaluronidase, and 0.05 mg mL^−1^ DNase I at 37°C for 120 min, then filtrated with 70 µm cell strainer. After removing RBCs, cells were purified furtherly. Moreover, the immune cells in lymph nodes and spleen tissues were collected as well. Cells were incubated with an anti‐CD16/32 antibody as an FC‐blocking agent to inhibit non‐specific binding before incubation with a fluorescence antibody. The population of CD8+INF‐γ+ and CD8+Granenzyme+ in CD3+ cells of tumor tissues and CD3+ CD8+ cells of lymph nodes, as well as MHCI+, MHCII+ and CD86+ in CD11c+ cells of lymph nodes were measured by flow cytometry and analyzed by the CytExpert software.

### 2.18 Systemic Toxicity Analysis

To evaluate the systemic toxicity of CM‐BPQDs, mice were sacrificed after being treated with PBS, BPQDs, and CM‐BPQDs every 2 days for four times, to collect the heart, liver, spleen, lung, and kidney, fixed in 4% PFA, and sectioned for H&E staining. H&E staining was performed by Wuhan Service Bio Technology Co.

### 2.19 Hemolysis

Blood was collected from healthy C57BL/6 mice into 1.5 mL tubes with anticoagulants. To separate the red blood cells, the blood was centrifugated at 1000 *g* for 10 min at 4°C. The RBC in the bottom layer was kept and washed with PBS for three times. CM‐BPQDs of different concentrations (5, 10, 20, and 40 µg mL^−1^) were incubated with 4% RBC in PBS for 4 h at 37°C. H_2_O was used as a positive control (100% hemolysis), while PBS was the negative control (0% hemolysis). After incubation, the supernatant was transferred into a new 96 wells to determine the OD value at 540 nm. The hemolysis index was calculated as following equation.

$$\begin{array}{cc} \mathrm{Hemolysis}\;\mathrm{index}\left(\%\right) & =\left(OD_{\mathrm{sample}}-OD_{\mathrm{PBS}}\right)/\left(OD_{H2O}-OD_{\mathrm{PBS}}\right)\\ & \;\times 100. \end{array}$$

### 2.20 Statistics Analysis

Descriptive and explorative analyses were performed using SPSS (Version 21.0.1, SPSS Inc., Armonk, NY, USA). All data were expressed as the mean ± standard error of the mean (SEM). D'Agostino‐Pearson test and Levene's test were performed to test the normality and the equality of variances of the data. Data were analyzed using one‐way ANOVA‐test in SPSS. The significance of the difference between the two groups was evaluated with Tukey test. *p* < 0.05 was set as the level of statistical significance.

## 3 Results and Discussion

### 3.1 The Physicochemical Properties of Protein were Critical for the BPNM‐PLK1 Interaction

**FIGURE 1:**
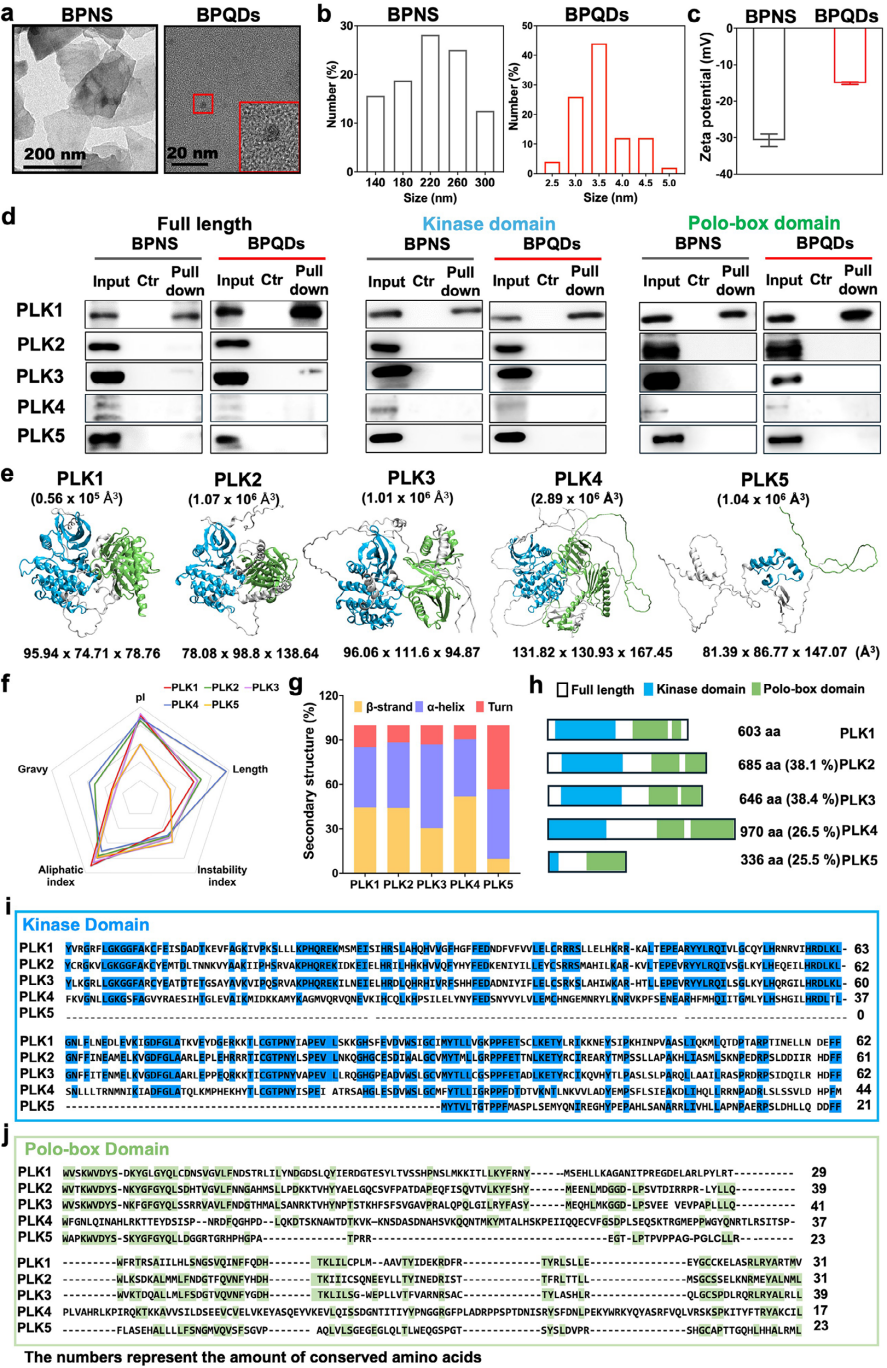
Characterization of BPNMs and proteins of the PLK family. (a–c) TEM images (a), size (b), and zeta potential (c) of BPNS and BPQDs. (d) The binding of PLK1/2/3/4/5‐Full length (FL), ‐Kinase domain (KD), and ‐Polo‐box domain (PD) with BPNS and BPQDs, assessed by pull‐down assay. (e) Three‐dimensional (3D) structure of the PLK family members. (f,g) The physicochemical properties (f), secondary structure (g) of the PLK family. (h) The key protein structures of the PLK family. The homology (%) was compared with PLK1. (i,j) Sequence alignment of kinase domain (KD) (i) and polo‐box domain (PD) (j) of the PLK family; The numbers represent the number of conserved amino acids.

To investigate whether the size of BPNMs affects the BPNM‐PLK1 interaction, two‐dimensional BPNS and zero‐dimensional BPQDs were used in the present study. BPNS showed a sheet‐like morphology with a mean lateral size of 224.88 ± 50.95 nm according to TEM (Figure 1a,b), and an average thickness of ≈6.2 nm determined by AFM (Figure S1). The average lateral size of BPQDs was 3.35 ± 0.52 nm according to the TEM images (Figure 1a,b). The zeta potential of BPNS was −30.7 ± 1.7 mV and BPQDs was −15.04 ± 0.34 mV in water (Figure 1c). Pristine BP has been proven to be hydrophobic [25].

Members of the PLK family share a similar architecture, consisting of an amino‐terminal catalytic domain (KD) and a unique carboxy‐terminal region (PD) with two or more polo boxes [16]. To explore whether BPNMs specifically bind with PLK1 in PLK family, we evaluated the binding ability of BPNMs to the PLK family proteins by pull‐down assay. The results showed that both BPNS and BPQDs could bind to PLK1 and its two functional domains (Figure 1d), which is in line with our previous results [23]. While only the full‐length PLK3 protein could weakly bind with BPNMs (Figure 1d). Nevertheless, no binding with BPNMs was observed for PLK2, PLK4, and PLK5 protein and their functional domains *in* *vitro* (Figure 1d). The results imply that PLK1 binds specifically with BPNMs, even within the highly conserved PLK family.

Our previous study identified that the intrinsic properties of BPNMs played a critical role in the BPNM‐PLK1 interaction [23]. Moreover, the properties of proteins also contributed to the NM‐protein interaction. To investigate the factors affecting the PLK1‐BPNMs specifically interaction, we compared the properties of the PLK proteins. The 3D structures of PLK proteins were shown in Figure 1e, the sizes were calculated and the order of the 3D size was PLK4 > PLK2 > PLK5 > PLK3 > PLK1. It implies that the 3D structure of PLK1 was compact, while its homologous proteins were loose. The 3D structure of PLK3 is mostly like PLK1 (Figure 1e). The pI of protein is defined as the pH value at which the net charge of the protein molecule is zero; thus proteins are positively charged at pH values lower than their pI and negatively charged at pH values higher than their pI [40]. The pI of PLK1 to 4 were 9.09, 8.52, 9.28, and 8.79, respectively and that of PLK5 was 5.99 (Figure 1f and Figure S2a). It implies that the surface charge of PLK1 to PLK4 is positive in the physiological environment, while PLK5 is negative. A stability index below 40 indicates that the protein is structurally stable, and above or equal to 40 indicates that the protein is unstable [41]. The stability index of PLK1 to PLK5 was 42.89, 50.91, 52.50, 49.29, and 58.05 (Figure 1f and Figure S2a), indicating that all five proteins of the PLK family were unstable. Another important parameter is the aliphatic index, which is the relative volume occupied by the aliphatic side chains in the protein and is a positive factor in improving thermal stability [42]. The aliphatic index of PLK1 to 5 were 90.40, 76.89, 85.80, 70.57, and 79.29, respectively (Figure 1f and Figure S2a). Moreover, the GRAVY is used to quantitatively analyze the hydrophilicity of proteins [43]. The positive values represent hydrophilic proteins and vice versa. GRAVY of PLK1 to PLK5 were −0.323, −0.514, −0.283, −0.577 and −0.315, meaning that the five proteins were hydrophobic proteins (Figure 1f and Figure S2a). Therefore, 3D structures, size, as well as physicochemical properties of PLK3 were found to be the most similar to those of PLK1 within the PLK family.

Furthermore, the structure of the protein is critical in defining the NM‐protein interface. As the Figure 1g and Figure S2b shown, the percentage of *β*‐sheet, *α*‐helix and turn were 44.5%, 40.7%, and 14.8% in PLK1, 44.2%, 44.2%, and 11.6% in PLK2, 30.4%, 56.5%, and 13.1% in PLK3, 51.9%, 38.5%, and 9.6% in PLK4 as well as 9.9%, 46.8%, and 43.3% in PLK5, respectively. A trend is observed across the PLK family, except for PLK5, where members exhibit a relatively high content of both β‐sheet and α‐helix. It is important to note that the complete secondary structure of full‐length PLK1 has yet to be experimentally elucidated. The data presented in Figure S2b are derived from AlphaFold predictions, which provide a single, most probable structural model based on the protein's amino acid sequence and available structural information. However, it is crucial to acknowledge the limitations of these predictions, as AlphaFold does not account for conformational dynamics or the influence of experimental conditions, such as pH, temperature, or solvent composition, on the protein's structural conformation [44]. Furthermore, the 1 to 5 of PLKs have 603, 685, 646, 970, and 336 amino acids, respectively. PLK1 consists of a KD and a PD with two polo boxes, and PLK2 and PLK3 are structurally like PLK1 with 38.1% and 38.4% homology, respectively (Figure 1h). PLK4 has an additional polo frame for homodimerization with a homology of 26.5% to PLK1, whereas PLK5 contains an inactive pseudo‐KD with a homology of 25.5% to PLK1 (Figure 1h) [45]. The high similarity was exhibited at the amino acid level in KD and PD of PLK1‐3 by the comparative analyses of the primary sequences of the PLK family, while PLK4 and PLK5 have a highly divergent sequence (Figure 1i,j) [16]. The physicochemical properties of the five proteins present a certain variation as influenced by the amino acid sequence. The sequence of PLK3 is most likely PLK1. This implies that the tertiary, secondary, and primary structure of PLK3 were most similar to those of PLK1 within the PLK family (Figure 1e–j), which facilitates the weak binding of PLK3 with BPNMs. This suggests that the physicochemical properties of proteins play a critical role in NM‐protein interactions.

### 3.2 The Amino Acids were Important for the BPNM‐PLK1 Interaction Energy

**FIGURE 2:**
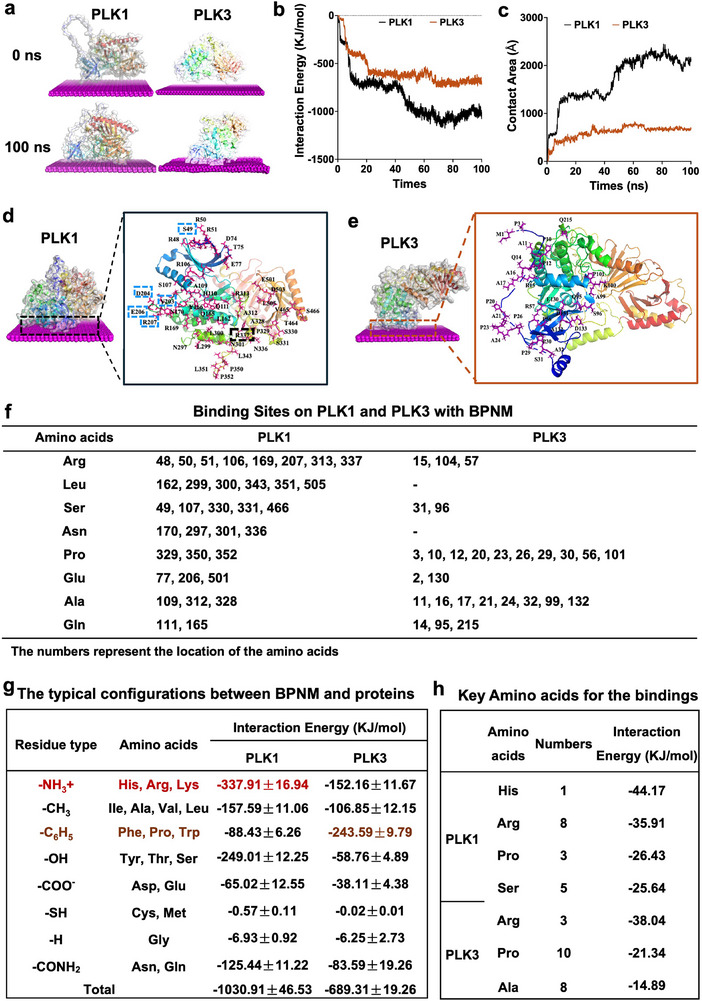
The binding of BPNMs with PLK1 and PLK3. (a) The snapshots of molecular dynamics (MD) simulation at initial state (0 ns) and final state (100 ns) for the interaction of BPNMs with PLK1 and PLK3. (b,c) Interaction energy (b) and contact area (c) in the MD simulation. (d,e) The interaction interface of BPNMs with PLK1 (d) and PLK3 (e). (f) The binding sites of BPNMs with PLK1 and PLK3. (g) The typical configurations and the related interaction energy between BPNM and PLK1. (h) The key amino acids contributing to the bindings of the BPNM‐PLK1 and BPNM‐PLK3.

In detail, MD simulations were conducted to explore the differences in interactions between BPNM‐PLK1 and BPNM‐PLK3, with three simulations performed for each protein. The results indicated that BPNM could bind to both PLK1 and PLK3 in all three simulation systems (Figure S3a,b). The average interaction energy was −890 KJ mol^−1^ in all three simulations of BPNM‐PLK1, while it was around −700 KJ mol^−1^ in BPNM‐PLK3 systems (Figure S3a,b). The results of the MD simulation were consistent with the pull‐down assay. One system from each interaction was selected for further comparison between BPNM‐PLK1 and BPNM‐PLK3 during 100 ns. The distance between BPNM and the protein gradually decreased with changes in the protein structure from 0 to 100 ns, indicating the binding of BPNM with both PLK1 and PLK3 (Figure 2a and Figure S4a). The interaction energy of both BPNM‐PLK1 and BPNM‐PLK3 was primarily due to van der Waals (vdW) contacts, with the energy for BPNM‐PLK1 being stronger than BPNM‐PLK3 (Figure 2b). In addition, the contact area of BPNM with PLK1 and PLK3 was 2136.64 and 693.54 Å, respectively (Figure 2c). The results indicated that the interaction BPNM‐PLK1 was stronger than that of BPNM‐PLK3.

We compared the binding interfaces of BPNM with PLK1 and PLK3 to identify differences driving their interactions. In the initial state, PLK1 and PLK3 are far from BPNM. At 8.5 ns, the residues Glu215, Arg11, Lys100, Lys217, and Pr101 contact BPNM. Following this time point, the number of contacting residues increased dramatically. By 80 ns, the binding between BPNM and PLK1 remains stable (Figure S5). As for PLK3, the binding with BPNM remains stable from 69.7 ns (Figure S5). In the final state, there are 43 binding sites in BPNM‐PLK1 interaction and 28 sites in BPNM‐PLK3 (Figure 2d,e). The BPNM‐PLK1 interaction involves six binding surfaces (Figure 2d) that facilitate stable interaction. However, the BPNM‐PLK3 interaction involves three small binding surfaces and one linear binding site (Figure 2e). The binding surfaces are composed of 4, 3, and 2 amino acids, respectively. The linear binding site, composed of 18 amino acids, is located at the N‐terminus of PLK3. Therefore, the interaction energy of BPNM‐PLK3 is weaker than that BPNM‐PLK1.

The key amino acids involved in BPNM‐PLK1 interaction include Arg (48, 50, 51, 106, 169, 207, 313, 337), Leu (162, 299, 300, 343, 351, and 505), Ser (49, 107, 330, 331, and 466), Asn (170, 297, 301 and 336), Pro (329, 350, and 352), Glu (77, 206, and 501), Ala (109, 312, and 328) and BPNM‐PLK3 interaction including Pro (3, 10, 12, 20, 23, 26, 29, 30, 56, and 10), Ala (11, 16, 17, 21, 24, 32, 99, and 132), Gln (14, 35, and 215), Ser (31 and 96), Glu (2 and 130) (Figure 2f). To further understand the role of specific amino acid residues in the binding of PLK1 and PLK3 to BPNM, we decomposed the interaction energies based on the side chain functional groups (including amino, methyl, benzene, hydroxyl, thiol, carboxyl, and hydrogen). The strongest energetic contributions to the BPNM‐PLK1 interaction were primarily from residues with amino side chains, such as histidine and arginine. In contrast, residues with benzene‐containing side chains, such as proline, dominated the interaction energy in the BPNM‐PLK3 interaction (Figure 2g). Subsequently, we calculated the average binding energies of the most contributive amino acids in contact with BPNM to further elucidate the key amino acids driving the interactions of PLK1 with BPNM. The average contact energies of histidine, arginine, and proline residues with BPNM were −44.17, −35.91, and −26.43 KJ mol^−1^, respectively, representing the highest values observed in PLK1 (Figure 2h). Additionally, the binding interface included one histidine, eight arginine, and three proline residues, with arginine contributing the most significantly to the overall energy in the BPNM‐PLK1 interaction. Similarly, the average contact energies of arginine, proline, and alanine were −38.04, −21.34, and −14.89 KJ mol^−1^, respectively, representing the highest values observed in PLK3 (Figure 2h). In this case, the binding interface included three arginine, 10 proline, and eight alanine residues, with proline contributing the most significantly to the overall energy in the BPNM‐PLK3 interaction. These findings suggest that arginine and proline are the key amino acids driving both the BPNM‐PLK1 and BPNM‐PLK3 interactions. The hydrophobic side chain of arginine can interact with the hydrophobic regions on the surface of BPNM, thereby enhancing the stability of the binding. Additionally, the long side chain of arginine provides greater potential for the binding of proteins and BPNM. Furthermore, the unique cyclic structure of proline allows it to form pockets or grooves, enhancing the binding between BPNM and PLK1 as well as PLK3 [46]. The stronger interaction between BPNM and PLK1 compared to PLK3 may be attributed to the higher contact energy and greater number of arginine residues in PLK1, likely due to the longer side chains of arginine. Therefore, arginine emerges as the most crucial amino acid driving the interaction energy between BPNM and PLK1.

### 3.3 The BPNMs Interact With the Function Domains of PLK1

**FIGURE 3:**
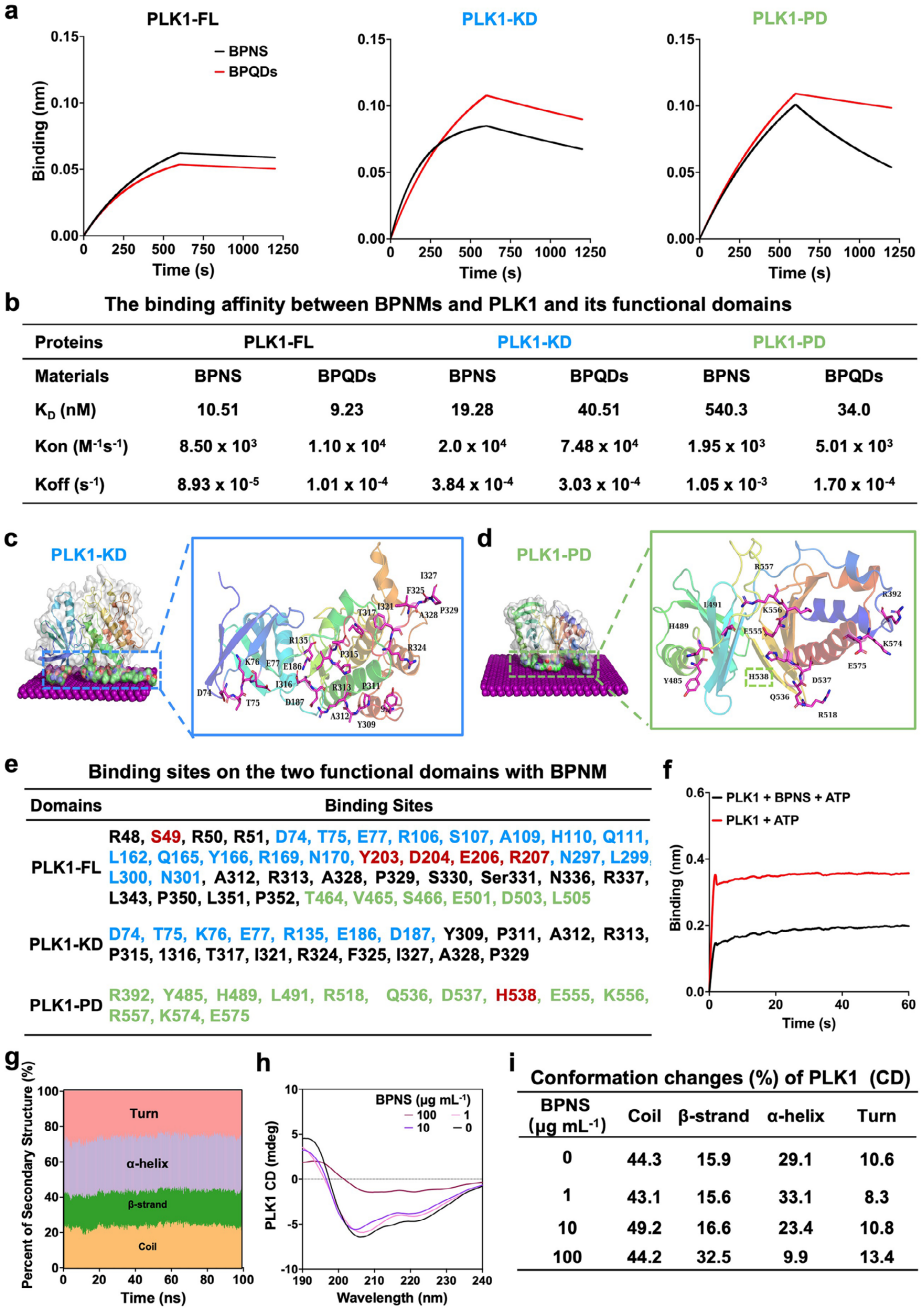
The interaction of BPNMs with PLK1 and its function domains. (a,b) The binding affinity of BPNS and BPQDs with PLK1‐Full length (FL), ‐Kinase domain (KD), and ‐Polo‐box domain (PD), determined by biolayer interferometry (BLI). (c,d) The interaction interface of BPNMs with PLK1‐KD (c) and ‐PD (d). (e) The binding amino acids in PLK1‐FL, ‐KD, and ‐PD by MD simulation. (f) Effect of BPNS on the binding of PLK1 with ATP assessed by the biolayer interferometry (BLI). (g) Conformational variation of protein between BPNMs and PLK1‐KD, and ‐PD during 100 ns of MD simulation. (h,i) Conformational variation of PLK1 after binding with BPNS, detected by circular dichroism (CD) spectra.

Further, we investigated the interaction of BPNMs with the full‐length and two functional domains of PLK1 in detail. First, the binding affinity was measured by BLI. As shown in Figure 3a,b, there was a strong binding affinity of PLK1‐FL, the kinase domain of PLK1 (PLK1‐KD), and the polo box domain of PLK1(PLK1‐PD) with BPNS and BPQDs. While the size of BPNMs have no effect on the binding affinity. Therefore, BPNMs represent a promising nano‐inhibitor of PLK1, exhibiting high specificity, strong binding affinity, and size independence.

The interaction interfaces of BPNM‐PLK1‐KD and ‐PD were investigated by MD simulation, respectively. PLK1 was divided into the KD (1‐345) and PD (346‐603) domains for the MD simulations (Figure S4b,c). The parameters of binding capacity were analyzed. The RMSD represents the change in protein conformation. An increased RMSD was observed (Figure S6a), indicating the obvious change of protein structure during MD simulation. The number of contact atoms was increased compared to the initial distance (Figure S6b). Additionally, the radius of gyration represents the compactness of the protein. The results showed the radius of gyration of PLK1‐KD and ‐PD also changed (Figure S6c), suggesting the compactness of the proteins was affected by binding to the BPNMs. Furthermore, the solvent accessible surface area of PLK1‐KD and ‐PD was decreased by binding of BPNMs (Figure S6d). The results fully confirmed the occurrence of BPNMs‐PLK1 interaction.

The binding energies were −3772.2 and −2468.9 KJ mol^−1^ for BPNM‐PLK1‐KD and ‐PD, respectively (Figure S7a–c). The binding energy of BPNM with the two functional domains of PLK1 is higher than that with the full‐length structure. This may be because these two independent domains are simpler and easier to bind than the full‐length structure. Therefore, it is essential to focus not only on interactions with protein domains but also on binding to the full‐length structure during drug development. In the interaction with PLK1‐PD, both arginine and lysine played a significant role (Figure S7a,c). Specifically, one lysine and three arginine residues were identified in the binding interface (Figure 3d,e), highlighting arginine as a key amino acid driving the BPNM‐PLK1‐PD interaction. These findings are consistent with those observed in PLK1‐FL, further emphasizing the critical role of arginine residues in mediating the interaction between BPNM and PLK1.

### 3.4 The PLK1‐BPNMs Interaction Affects the Protein Function of PLK1

Furthermore, the binding sites of BPNM to PLK1‐KD and ‐PD were listed in Figure 3c–e. The hydrophobic amino acids of PLK1‐KD contributed the binding energy including Y309, P311, A312, P315, I316, I321, F325, I327, A328, and P329 by their large hydrophobic side chain. The common binding sites in the PLK1‐FL and PLK1‐KD systems were D74, D75, E77, A312, R313, I327, A328, and P329. The binding sites in the PLK1‐PD were R392, Y485, H489, L491, R518, Q536, D537, H538, E555, K556, R557, K574, and E575 (in Figure 3c,e). H538 (highlighted in Figure 3e) was the critical amino acid in substrate recognition and binding [16]. The binding sites of BPNM with PLK1‐FL, specifically S49, Y203, D204, E206, R207, and R337 (highlighted in red in Figure 3e), are essential for the functional activity of PLK1. S49 has been identified as a novel phosphorylation site within the kinase domain, contributing to full activation of PLK1 during mitosis [47]. The binding sites Y203, D204, E206, and R207 are located within the activation‐loop, where the conserved residue (T210) serves as the primary site for PLK1 activation. The conformation of the flexible activation loop is critical for PLK1's binding to its substrates [48]. In addition, R337 is situated in the D‐box region, which is recognized and ubiquitinated by the anaphase‐promoting complex/cyclosome for PLK1 degradation [21]. Mutation at the R337 residue renders PLK1 non‐degradable, resulting in a delay in mitosis exit [49]. Then BLI was used to evaluate whether the BPNM‐PLK1 interaction affected the substrate binding of PLK1. The data showed that the binding of PLK1 with ATP was decreased in the presence of BPNS (Figure 3f). The results imply that BPNM can specifically bind to and disrupt the function of PLK1, highlighting it as a promising PLK1 inhibitor.

MD simulation was further used to investigate whether the BPNM‐PLK1 interaction affected the protein structure of PLK1. The amounts of salt bridges, π–π stacking interactions were increased in both of PLK1‐KD and PLK1‐PD after binding with BPNMs (Figure S8a). The results indicated that the interactions of amino acids within the protein change along with the binding with BPNM, especially the number of hydrogen bonds and salt bridges, which are very important for maintaining protein folding, structure, and function. Furthermore, the structure of proteins may change after interaction with a NM, since such an alteration can have a profound effect on protein function [50]. Therefore, it is vital to determine the structural changes affected by NMs. We explored the changes in the secondary structure of the PLK1 induced by BPNMs. The percentage of coil, *β*‐sheet, *α*‐Helix, and turns in PLK1 was significantly changed by BPNM in silico (Figure 3g and Figure S8b). Meanwhile, BPNM significantly reduced the number of hydrogen bonds after interacting with PLK1‐KD and PLK1‐PD. The percentage of turn and coil changed in PLK1‐KD, and that of *β*‐sheet, *α*‐Helix, and turns was significantly changed by BPNS. Furthermore, CD spectra were performed to assess the change of PLK1 conformation caused by BPNS. The percentage of *α*‐helix, *β*‐helix, and turn in the PLK1 protein was all changed after binding with BPNS (1, 10, and 100 µg mL^−1^) compared to the control (Figure 3h,i). The results demonstrated that the binding of BPNMs interfered with the PLK1 conformation.

### 3.5 Cell Membrane Coating Improves the Stability and Tumor‐Targeting Capacity of BPNMs

**FIGURE 4:**
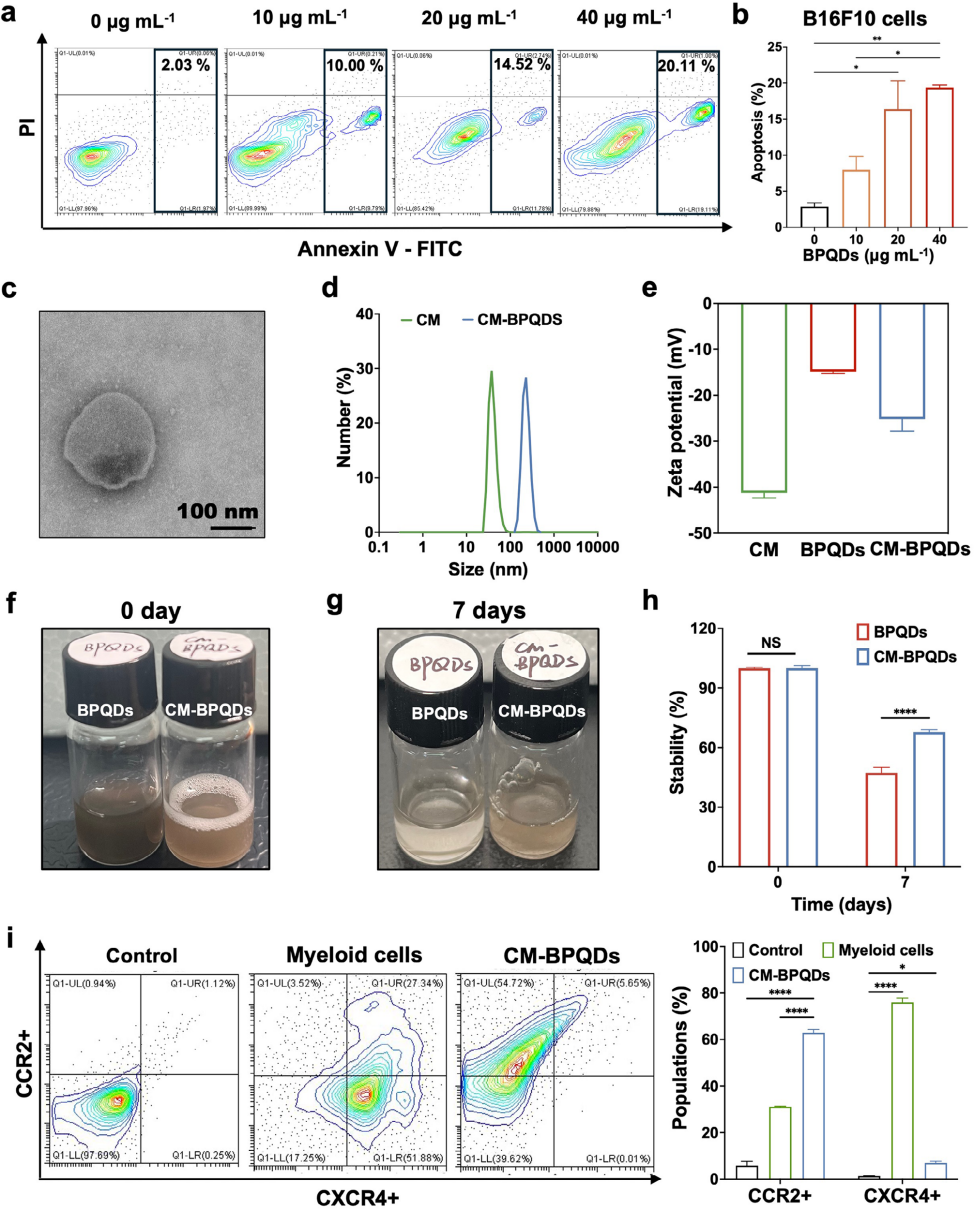
Characterization of CM‐BPQDs. (a,b) Flow cytometry analysis of apoptosis of B16F10 cells induced by BPQDs. (c) TEM image of CM‐BPQDs. (d) Size of CM and CM‐BPQDs. (e) Zeta potential of CM, BPQDs and CM‐BPQDs. (f–h) Photographs (f,g) and the variation of absorption intensity of the maximum peak (h) of the sample of BPQDs and CM‐BPQDs in water after storing 7 days. (i) CRR2+ and CXCR4+ on CM‐BPQDs and myeloid cells. Data are presented as mean ± SEM. *p < 0.05, **p < 0.01, ****p < 0.001 compared with control group (b, h, i).

Our previous study already verified that BPNS significantly inhibited tumor growth *in* *vivo* [23]. With a smaller size, larger specific surface area, and higher band gap than conventional 2D BP nanosheets, BPQDs could enhance the stability of physiological media and be more easily endocytosed into cells without disrupting membrane integrity [51]. Importantly, the binding of BPNMs to PLK1 was size independent. In such a scenario, it is necessary to explore the chemotherapy efficiency of BPQDs. As shown in Figure 4a,b, the cell death of B16F10 cells was significantly induced by 10, 20, and 40 µg mL^−1^ BPQDs in a dose‐dependent manner, indicating BPQDs possess effective antitumor activity and hold potential as a chemotherapeatic agent.

However, disadvantages of BPQDs are comprising its chemotherapy efficiency, such as the instability and poor tumor targeting [25]. Cell membrane coated nanomaterials (CNMs) could be a powerful approach to protect BPQDs  from degradation in biological fluids [27]. Furthermore, myeloid‐derived cells have the capacity to infiltrate to the tumor microenvironment (TME), which could be considered as an effective vehicle to targeting deliver drugs for cancer treatment [28]. Therefore, combination of the CNMs approach and the targeting capacity of myeloid‐derived cells presents a promising solution to enhance the chemotherapy efficiency of BPNMs. We coated cell membranes of myeloid‐derived cells onto BPQDs to construct cell membrane‐coated BPQDs (CM‐BPQDs) and explored the chemotherapeutic effect in vivo. The CM‐BPQDs were confirmed by FE‐TEM as shown in Figure 4c. The size of the cell membrane (CM) and CM‐BPQDs were respectively 244.6 ± 40.95 nm and 367.7 ± 15.62 nm, determined by DLS (Figure 4d). The zeta potential of CM and CM‐BPQDs were −41.2 ± 1.16 mV and −25.17 ± 2.66 mV, respectively (Figure 4e).

Furthermore, the BPQDs and CM‐BPQDs were dispersed in water and exposed to air for 7 days to assess their stability by monitoring the UV‐vis absorption spectra. As shown in Figure 4g,h, and Figure S9, the color of the BPQDs significantly lightened after 7 days, indicating that most of the BPQDs had degraded, with degradation rates around 53%. In contrast, the color of the CM‐BPQDs solution only slightly lightened, with degradation rates around 33%. It implies that the strategy of cell membrane coating effectively decreases the degradation rates of BPQDs.

Cell membrane preparations and CM‐BPQDs were evaluated to determine whether depriving and coating processes with the cell membrane would affect its integrity and the activity of the membrane proteins. As shown in Figure S10, the proteins of CM‐BPQDs were similar to cell membrane proteins, indicating that membrane proteins were mostly retained by CM‐BPQDs. Chemokine receptors such as CCR2 and CXCR4 play crucial roles in facilitating tumor growth and immune suppression by promoting cell migration and retention within the TME [38]. Specifically, CRR2 and CXCR4 are important chemokine receptors on myeloid cells that enhance their migration and retention in the TME. CCR2, primarily expressed on the surface of monocytes and other myeloid cells, is critical for the chemotactic migration of these cells toward sites of TME in response to its ligand, CCL2 (monocyte chemoattractant protein‐1 or MCP‐1) secreted by tumors [52]. Meanwhile, CXCR4 is another crucial chemokine receptor involved in the regulation of myeloid cell trafficking, particularly in the context of tumor biology. This receptor binds to its primary ligand, CXCL12 (also known as stromal cell‐derived factor‐1 or SDF‐1), which is abundantly expressed in many TME [53]. The chemotaxis of myeloid cells toward tumor sites is facilitated by the activation of these receptors, enabling myeloid cells to effectively navigate and respond to the TME. To evaluate the tumor‐targeting capacity of CM‐BPQDs, we detected CCR2 and CXCR4 on CM‐BPQDs using flow cytometry. As shown in Figure 4i, both CCR2 and CXCR4 on CM‐BPQDs are comparable to levels found on native myeloid cells. This strongly indicates that the myeloid cell membrane was successfully encapsulated and that it retained key targeting molecules, further supporting the potential of CM‐BPQDs for targeted delivery within the TME.

### 3.6 Anti‐Tumor Effects of CM‐BPQDs *i* *n* *v* *ivo*

**FIGURE 5:**
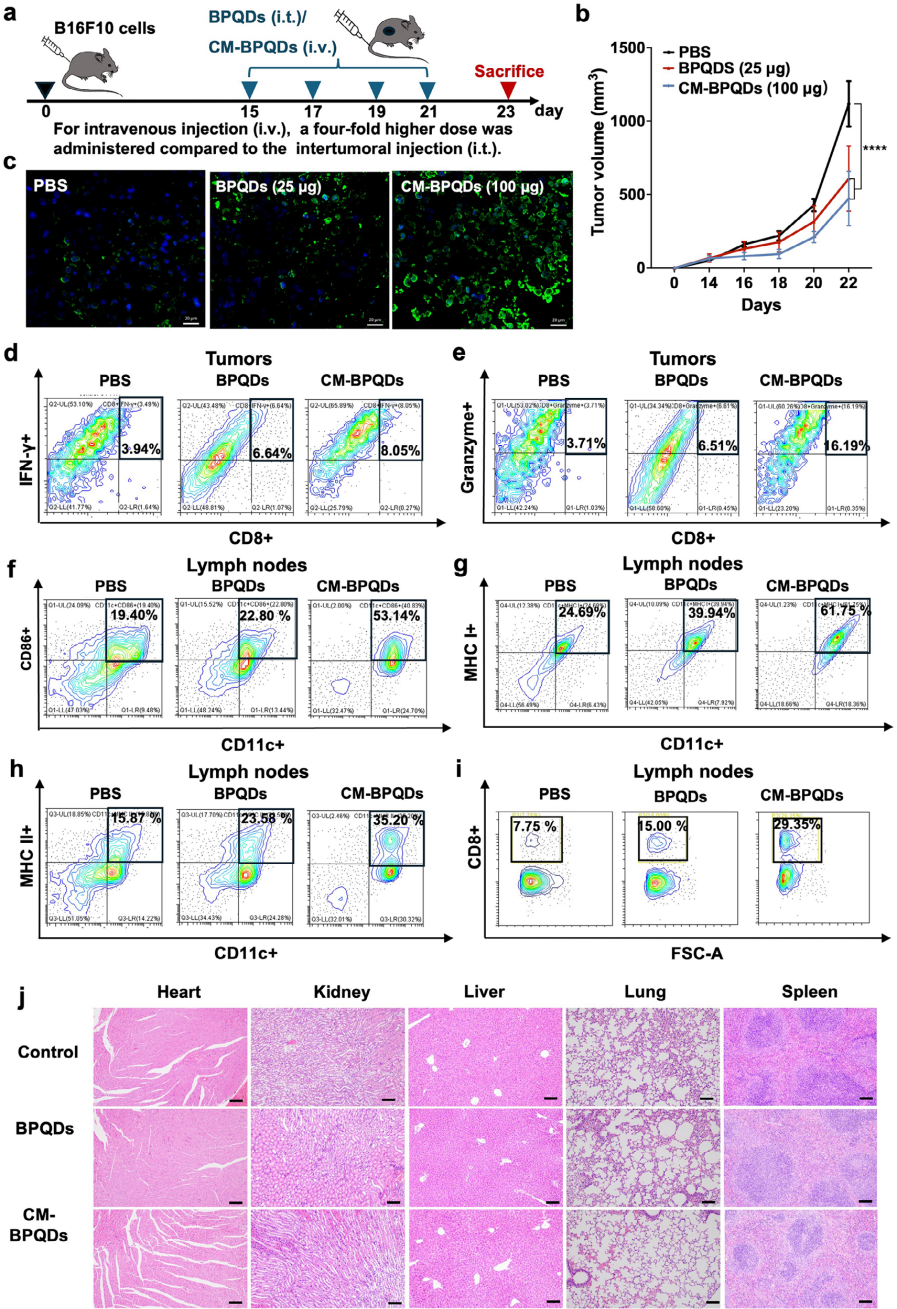
Anti‐tumor effect of CM‐BPQDs in vivo. (a) Timeline of anti‐tumor treatment. Melanoma bearing mice were administrated with BPQDs (25 µg) by intertumoral injection (i.t.) and CM‐BPQDs (100 µg) by intravenous injection (i.v.) every other day for four times. (b) Tumor growth curves during the treatment process. Data are presented as mean ± SEM. ****p < 0.0001, compared with the control group. (c) Image of tumor tissues stained with TUNEL. (d) CD8+IFN‐γ+ and (e) CD8+Granzyme+ in CD3+ cells in tumors after BPQDs and CM‐BPQDs treatments in tumor‐bearing mice. (f–h) CD86+ (f), MHCI+ (g), and MHCII+ (h) in CD11c+ cells and (i) CD8+ in CD3+ cells in lymph nodes after BPQDs and CM‐BPQDs treatments in melanoma‐bearing mice. (j) Systemic toxicity analysis in vivo. H&E sections of major organs, including the heart, kidney, liver, lung, and spleen of mice after treatments. The scale bar is 100 µm.

Our previous study evaluated the anti‐tumor effects of BPNS by intratumorally injection every other day, four times, and the results showed a significant decrease in tumor size [23]. To evaluate the anti‐tumor effect of CM‐BPQDs via systematic administration, we treated melanoma bearing mice with CM‐BPQDs (100 µg) by intravenous injection (i.v.) every other day for a total of four injections (Figure 5a). For comparison, BPQDs (25 µg, the same concentration as in our previous study) were intertumoral injection (i.t.), because the dose is normally increased 2–5 times when systematic administration is performed [39b]. The results showed the tumor size significantly decreased in the BPQDs group and the CM‐BPQDs group compared with the control (****p < 0.0001), while no difference was found among the BPQDs and the CM‐BPQDs groups (Figure 5b). As shown in Figure S11, the control group shows an intact tumor structure with uniformly arranged cells, while both the BPQDs and CM‐BPQDs groups exhibit significant tumor disruption. These histological findings support our quantitative data on tumor volume reduction and visually demonstrate the effectiveness of the treatment in damaging tumor tissue. Furthermore, tumor cell death was observed in the BPQDs and CM‐BPQDs groups (Figure 5c). These results proved that the chemotherapeutic effects of CM‐BPQDs (100 µg, i.v.) is comparable to that of BPQDs (25 µg, i.t.). Therefore, the cell membrane coating strategy presented a promising approach to overcome the fast degradation rate of potential drugs for effective therapeutics via systematic administration *in* *vivo*.

In addition, we tested the immunomodulating effect of BPQDs and CM‐BPQDs in TME. Significantly increased (**p < 0.01) population of CD8+ IFN‐γ+ in CD3+ cells was observed in tumor tissues of CM‐BPQDs group compared with both control and BPQDs group (Figure 5d and Figure S12a). The population of CD8+ Granzyme+ in CD3+ cells was also significantly increased (**p < 0.01) in tumor tissues after CM‐BPQDs treatment (Figure 5e and Figure S12b), indicating an enhanced function of cytotoxic T lymphocytes in tumors. Similar results were reported in BP‐based cancer immunotherapy, suggesting an effective tumor cell death caused dendritic cells (DCs) recruitment for tumor‐specific CD8+ T cells activation [54]. BPQDs can activate immune pathways, such as NF‐κB and pattern recognition receptors (PRRs), which play a crucial role in initiating immune responses. The activation of these pathways promotes the maturation of DCs, thereby enhancing their antigen‐presenting capability and, in turn, improving T cell activation [55]. This mechanism likely accounts for the observed enhancement in T cell function after CM‐BPQD treatment.

The activation of specific T cells has been confirmed in the above study; thus, further evaluation of DC activation was required in the nearby lymph nodes. Significantly increased population of CD86+ CD11c+ cells was found in lymph nodes after CM‐BPQDs treatment (*p < 0.05; Figure 5f and Figure S12c), implying enhanced DC maturation and reflux to the nearby lymph nodes. Furthermore, the MHC I+ and MHC II+ expression on CD11c+ DCs was significantly increased in lymph nodes (*p < 0.05; Figure 5g and Figure S12d; Figure 5h and Figure S12e). It suggestes that effecient presentation of tumor‐associated antigen derived from apoptotic tumor cells in lymph nodes promoted the activation of specific CD3+CD8+ T cells (*p < 0.05, **p < 0.01; Figure 5i and Figure S12f), there by contributiong to the antitumor immune response.

### 3.7 The Biosafety Assessment and Systemic Toxicity Analysis

Safety is a key issue for the successful clinical applications of CM‐BPQDs [56], thus, the biosafety and systemic toxicity were assayed. As shown in Figure S13a, no cytotoxicity was observed in primary HEKa cells after treating with CM‐BPQDs for 24 h, which suggested CM‐BPQDs were safe within the doses for normal cells. The reason why CM‐BPQDs cannot kill HEKa cells effectively is that PLK1 is normally overexpressed in cancer cells rather than in normal cell types [17b]. In the meantime, no hemolysis was observed after incubating CM‐BPQDs with blood red cells for 2 h (Figure S13b), indicating the biocompatibility of CM‐BPQDs.

To assess the systemic toxicity of CM‐BPQDs *in* *vivo*, H&E staining was used to analyze the toxicity to major organs, including heart, liver, spleen, lung, and kidney. As shown in Figure 5j, no abnormalities were observed in the heart, liver, spleen, lung, and kidneys by CM‐BPQDs. The results suggested that CM‐BPQDs were not toxic at the concentration used *in* *vivo* for tumor treatment.

This study demonstrates that BPNMs specifically bind to PLK1 and its two functional domains within the PLK family, exhibiting high binding affinity and size independence. These results are consistent with our previous study [23]. Notably, oxidation of BP enhances its interaction with PLK1‐PD, as oxygen atoms can occupy the edge and surface sites of BP, facilitating phosphate groups formation [57]. This further confirms that the specific binding of the BPNMs to PLK1 is driven by the intrinsic physicochemical properties of BPNMs.

In detail, BPNMs bind with PLK1 and its functional domains, as well as with PLK3. Whereas no binding with BPNMs was observed with other members of the PLK family. The physicochemical properties of PLK1 and PLK3 played important roles in PLK1‐BPNM and PLK3‐BPNM interactions, for instance, the electrostatic force with positive surface charge, the hydrophobicity, and the protein 3D structure and sizes versus its homology proteins in the PLK family. Higher hydrophobicity can promote the binding of NMs and proteins [58]. Both pristine BPNMs as well as PLK1 are hydrophobic. The interactions between hydrophobic NMs and proteins could lead to conformational changes of proteins, as the hydrophobic core within the protein might become exposed to the polar solvent environment through hydrophobic interactions [12, 50]. Furthermore, the energy of PLK1‐BPNM interaction mainly contributed to the vdW contacts, which was driven by the large hydrophobic side chains of hydrophobic amino acids. The hydrophobic amino acids, especially arginine, played a key role in mediating the BPNM‐PLK1 interaction. Thereby, the specific binding of PLK1 to BPNMs reflects a mutually selective interaction, driven by the optimal physicochemical properties of PLK1 and the intrinsic bioactivity of BPNMs. This work indicates that BPNMs can be a promising and specific inhibitor of PLK1 for cancer chemotherapy.

Cell membrane coating strategy could be used to protect from rapidly degradable NMs in biological fluids. Myeloid cells hold many chemokine receptors, which could be attracted by the chemokines secreted by the TME, thereby enhancing the tumor‐targeting capacity. Our results confirmed the therapeutic effect CM‐BPNMs *in* *vivo* after systemic administration, highlighting the tumor‐targeting capacity of the myeloid cell membrane coating strategy.

In summary, this work provides significant insights into the molecular basis of BPNM‐PLK1 interaction, revealing a promising avenue for developing molecular targeting NMs for cancer therapies. The unique physicochemical properties of BPNMs, particularly their specific binding to PLK1 and its functional domains, offer a novel method for targeted oncotherapy. This PKL1‐targeting specificity of BPNMs presents significant potential for developing more effective and less toxic nano‐based cancer treatments. Additionally, the application of a cell membrane coating strategy further enhances the therapeutic efficacy of BPNMs as PLK1‐specific inhibitors. This innovation addresses key limitations of BPNMs, such as rapid degradation, while improving tumor‐targeting capacity.

Despite their promising potential, several challenges must be addressed for the clinical translation of BPNMs for cancer therapy. First, ensuring robust therapeutic efficacy across diverse patient populations is a critical hurdle. This requires strategies to address variations in PLK1 expression levels and optimize dosing regimens. Secondly, improving biodistribution is vital for maximizing therapeutic impact, which necessitates enhancing tumor penetration and developing real‐time imaging techniques to track BPNM distribution. Additionally, long‐term safety is a major concern for clinical application, necessitating comprehensive toxicology studies, evaluation of biodegradation and clearance, and assessment of potential immunogenicity. Moreover, ensuring reproducibility in the cell membrane coating process is crucial, which involves maintaining consistent properties and standard cell membrane sources. To proceed with future clinical translation of BPNMs for cancer therapies, further development should focus on optimizing the functionality of BPNMs for enhanced targeting; the development of personalized treatment strategies based on biomarkers predictive of PLK1 inhibition response will enable more precise and effective therapies tailored to individual patients. These efforts will pave the way for the successful clinical translation of BPNMs as the next‐generation cancer therapies.

## 4 Conclusion

Our study demonstrated that the BPNM‐PLK1 interaction is driven by the physicochemical properties of both protein and BPNMs. It elucidated the mechanism by which BPNMs act as an effective PLK1 inhibitor for cancer chemotherapy and offered a practical solution for targeted cancer treatment using myeloid cell membrane‐coated BPNMs. In conclusion, this study provides essential insights into BPNMs as PLK1 inhibitors and presents a viable strategy for their application in cancer treatment, thereby paving the way for the clinical use of BPNMs as cancer chemotherapeutics.

## Author Contributions

**Fangfang Liu**: study design, experimental and investigation process, data analysis, data presentation, original draft, manuscript editing. **Zhong‐Da Li**: animal experiment, flow cytometry, data analysis, data presentation. **Yanqiao Zeng**: plasmids construction, animal experiment, and protein purification. **Xiaofeng Wang**: MD simulation, data interpretation, and manuscript editing. **Yingnan Liu**: BLI, animal experiment, and flow cytometry. **Qi Li**: BLI, plasmids construction, animal experiment. **Wenhe Luo**: flow cytometry, data analysis, and data presentation. **Xiaoman Suo**: protein purification and animal experiment. **Yaqing Xu**: animal experiment and characterization of nanomaterials. **Feng Yuan**: animal experiment, flow cytometry, and data analysis. **Dan Zhang**: protein purification and nanomaterials stability. **Wuqiong Zhang**: flow cytometry. **Shengyong Geng**: synthesis of nanomaterials. **Xue‐Feng Yu**: synthesis of nanomaterials. **Guofang Zhang**: study design, data analysis, presentation and interpretation, manuscript editing. **Yang Li**: conceptual, supervising, study design, data analysis, presentation and interpretation, manuscript editing.

## Conflicts of Interest

All authors declare that the research was conducted in the absence of any commercial or financial relationships that could be construed as a potential conflict of interest.

## Supporting information




**Supplementary File 1**: exp270090‐sup‐0001‐SuppMat.docx.

## Data Availability

The data that support the findings of this study are available from the corresponding author upon reasonable request.

## Appendix A Supplementary data

Supplsementary tables and figures include the AFM image of BPNS, charaterization of proteins in the PLK family, three runs of MD simulation between BPNMs binding to PLK1 and PLK3 in silico, snapshots of BPNM binding with PLK1 during 100 ns in silico, the binding of BPNM to amino acid residues in PLK1 and PLK3 in different stage during 100 ns, BPNM binding with PLK1‐KD and ‐PD in silico, typical interaction configuration between BPNM with PLK1‐KD and ‐PD, conformation variation of PLK1‐KD and ‐PD after binding with BPNMs, the UV–vis absorption spectra of BPQDs and CM‐BPQDs in water at day 0 or after 7 days, proteins on cell membranes and CM‐BPQDs, images of tumor tissues stained with HE as well as flow cytometry analysis of immune response in tumor tissues and lymph nodes after systematic administration of CM‐BPQDs in melanoma‐bearing mice, as well as the safety of CM‐BPQDs in vitro.
