# Supplementary material for: Target Binding of Black Phosphorus Nanomaterial to Polo‐Like Kinase 1 for Cancer Chemotherapy: A Mutual Selection of Nanomaterial and Protein
Source: Exploration (Beijing). 2025 Sep 26;5(6):20240143. doi: 10.1002/EXP.20240143 (PMC12752640; doi:10.1002/EXP.20240143)
Supplement: Supplementary file 1 — exp270090‐sup‐0001‐SuppMat.docx. [file EXP2-5-20240143-s001.docx]

**Target binding of black phosphorus nanomaterial to polo-like kinase 1 for cancer chemotherapy: a mutual selection of nanomaterial and protein**

Fangfang Liu^1,2,#^, Zhong-Da Li^1,2,#^, Yanqiao Zeng^1,2,#^, Xiaofeng Wang^1,2^, Yingnan Liu^1,2,3^, Qi Li^1,2,4^, Wenhe Luo^1,2^, Xiaoman Suo^1,2^, Yaqing Xu^1,2^, Feng Yuan^1,2^, Dan Zhang^1,2^, Wuqiong Zhang^1,2^, Shengyong Geng^5^, Xue-Feng Yu^5,6^, Guofang Zhang^1,2,^*****, Yang Li^1,2,6,^*****

^1^Laboratory of Inflammation and Vaccines, Shenzhen Institute of Advanced Technology, Chinese Academy of Sciences, Shenzhen 518055, China

^2^Laboratory of Immunology and Nanomedicine & China-Italy Joint Laboratory of Pharmacobiotechnology for Medical Immunomodulation, Shenzhen Institute of Advanced Technology, Chinese Academy of Sciences, Shenzhen 518055, China

^3^Department of Biosciences and Medical Biology, Division of Allergy andImmunology, Paris Lodron University of Salzburg, A-5020 Salzburg, Austria

^4^Universitat Autònoma de Barcelona (UAB), Campus UAB, Bellaterra, 08193 Barcelona, Spain

^5^Materials and Interfaces Center, Shenzhen Institutes of Advanced Technology, Chinese Academy of Sciences, Shenzhen 518055, China

^6^Key Laboratory of Biomedical Imaging Science and System, Chinese Academy of Sciences, Shenzhen 518055, China

These authors contribute equally: Fangfang Liu, Zhong-Da Li, Yanqiao Zeng

*****Correspondence:

Guofang Zhang: gf.zhang@siat.ac.cn

Yang Li: yang.li@siat.ac.cn


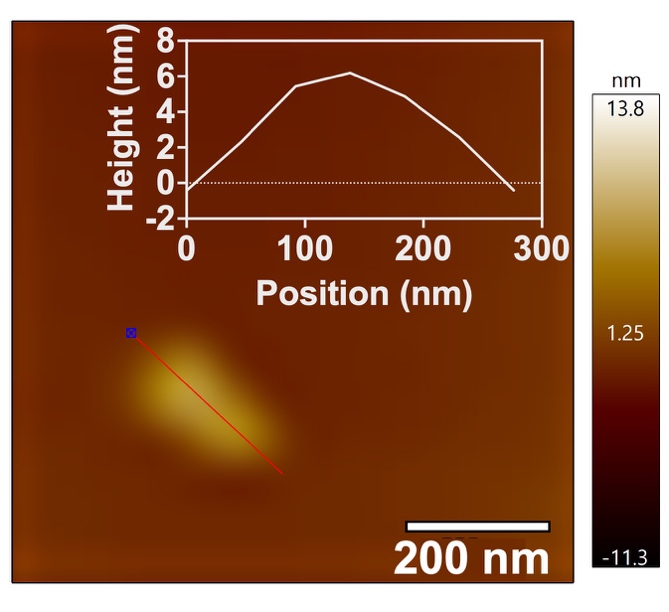


**Figure S1. AFM image of BPNS**. Inset data represents the height measured on the red line.


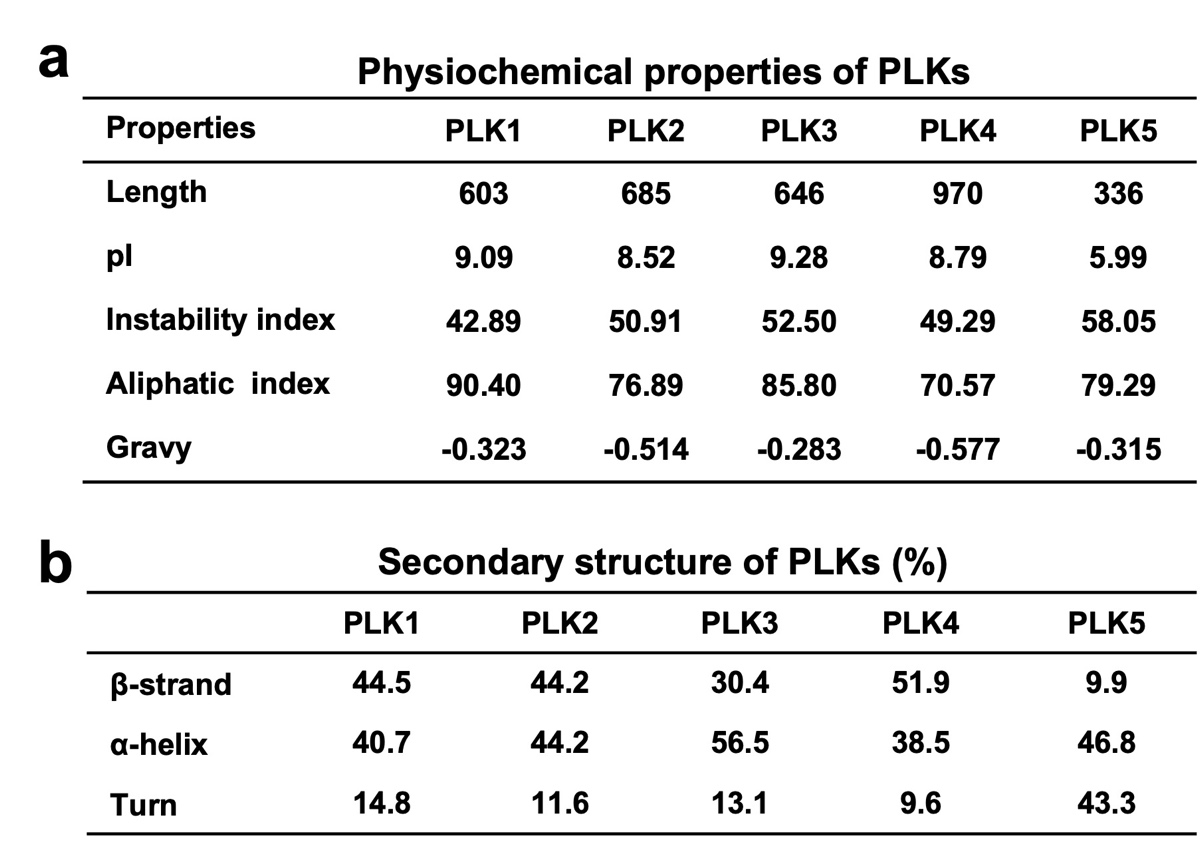
**Figure S2. Characterization of proteins in the PLK family.** a-b) The physicochemical properties (a, corresponding to Figure. 1e), secondary structure (b, corresponding to Figure. 1f) of the PLK family.


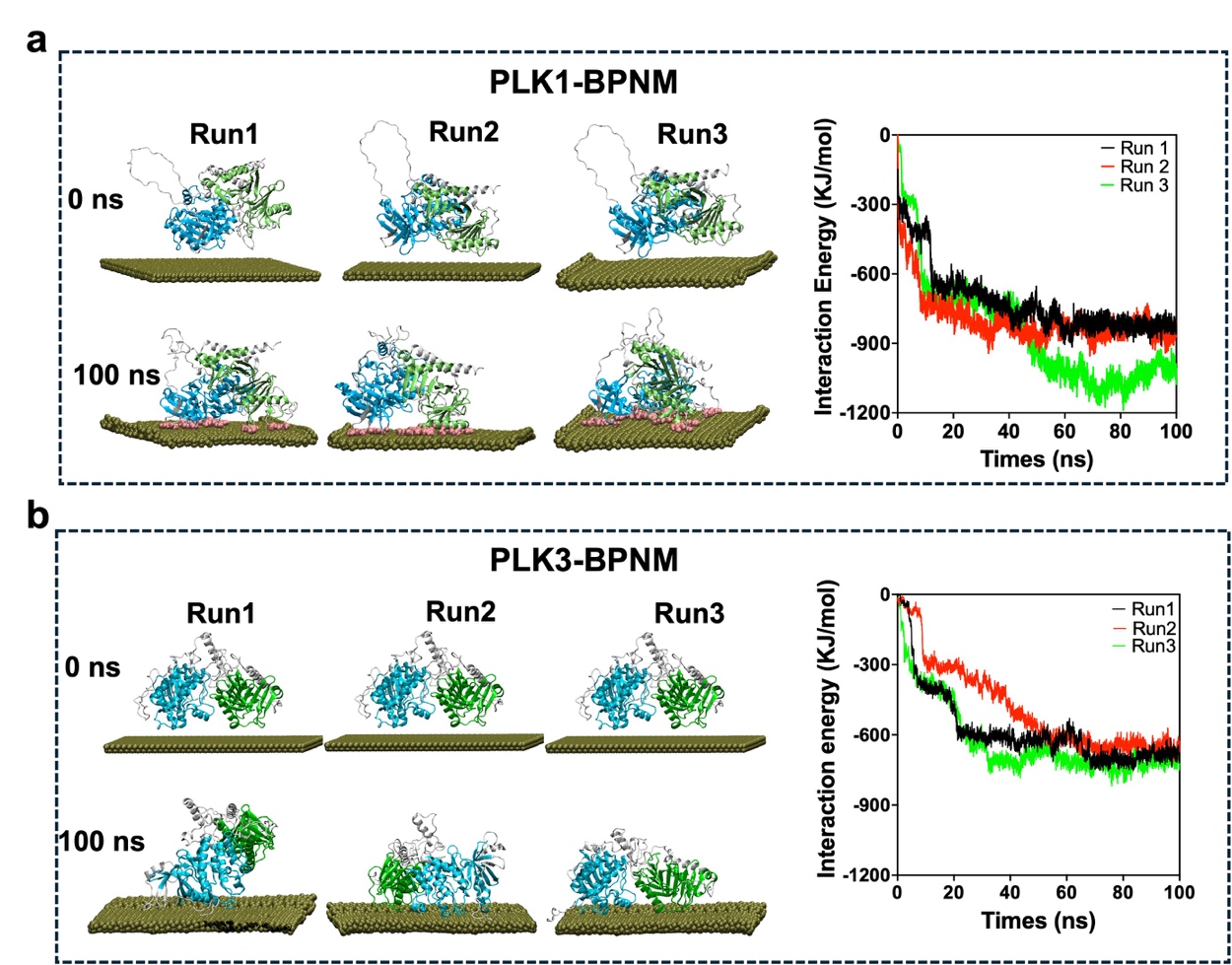


**Figure S3. Three runs of MD simulation between BPNMs binding to PLK1 and PLK3 *in silico.* a-b)** Snapshots from molecule dynamic (MD) simulations at initial and final stages, illustrating the interactions of BPNM with PLK1 and PLK3, along with corresponding interaction energy.

**
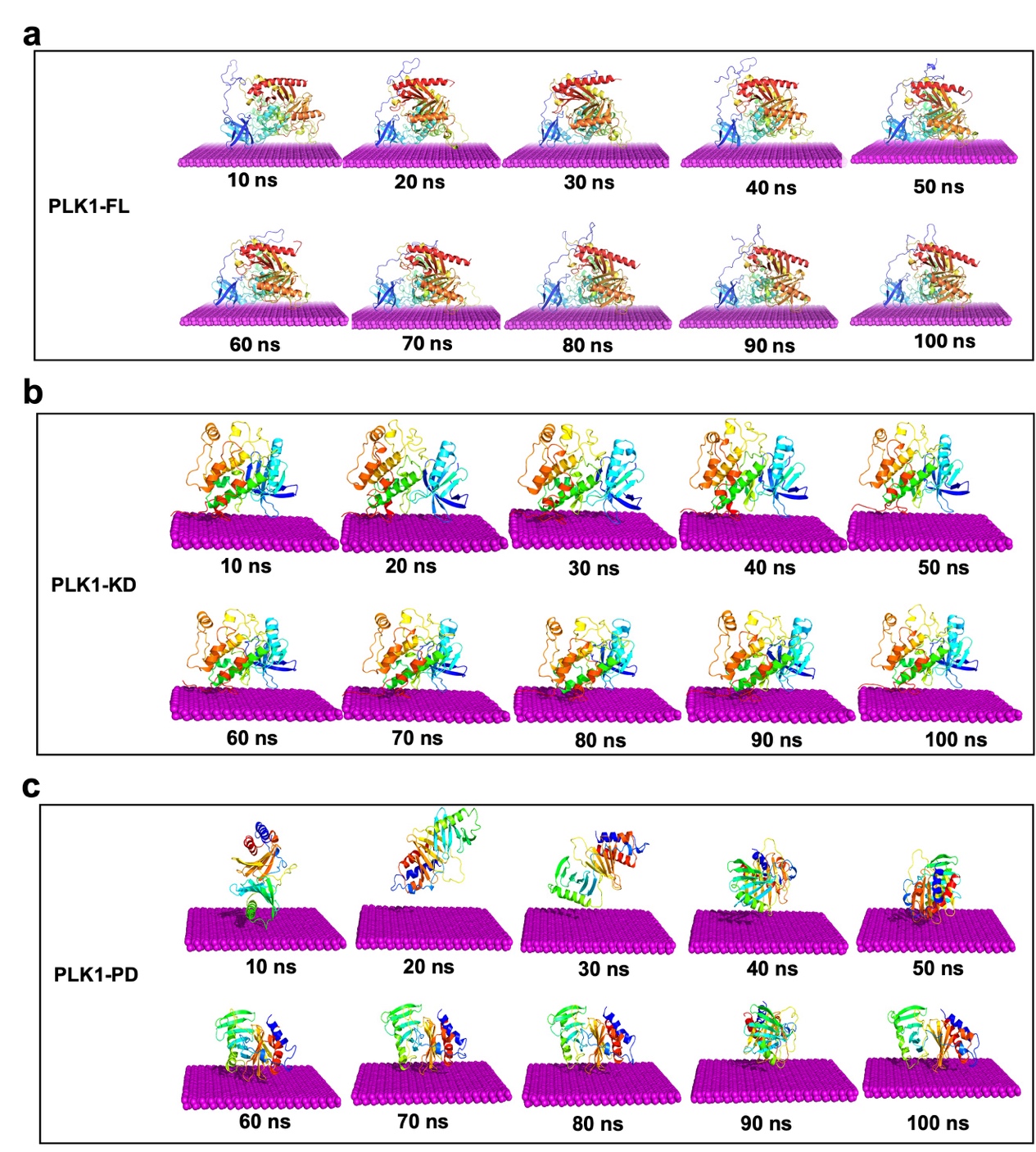
**

**Figure S4. Snapshots of BPNMs binding with PLK1 during 100 ns *in silico.* a-c)** Snapshots of molecule dynamic (MD) simulation at different times for the interaction of BPNM with PLK1.


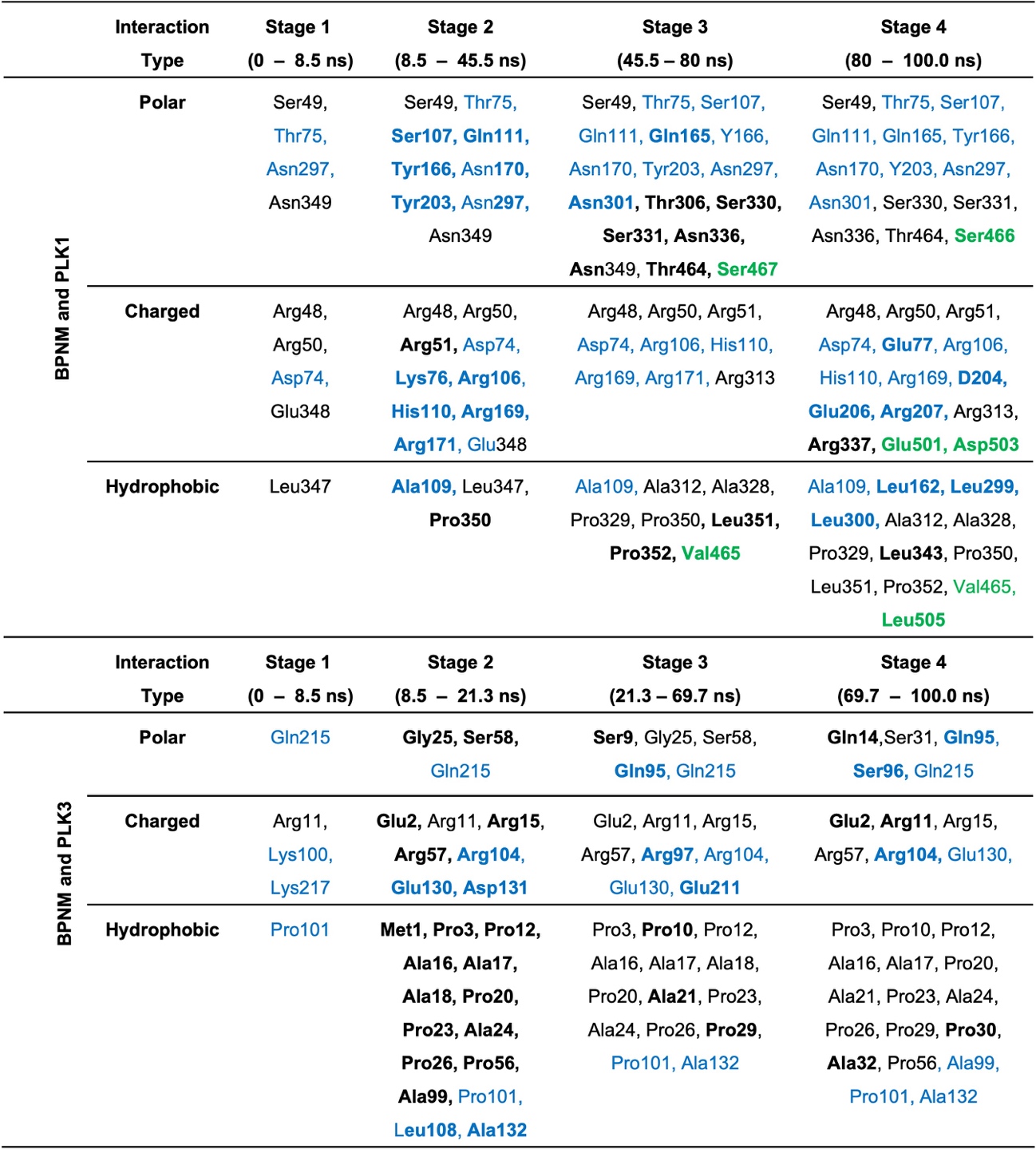


**Figure S5.** **The binding of BPNM to amino acid residues in PLK1 and PLK3 in different stage during 100 ns.** The amino acids highlighted in blue are located in the KD (kinase domain), while those in green are found in the PD (phosphatase domain). The new amino acids, indicated in bold, represent the new bindings identified in this stage.


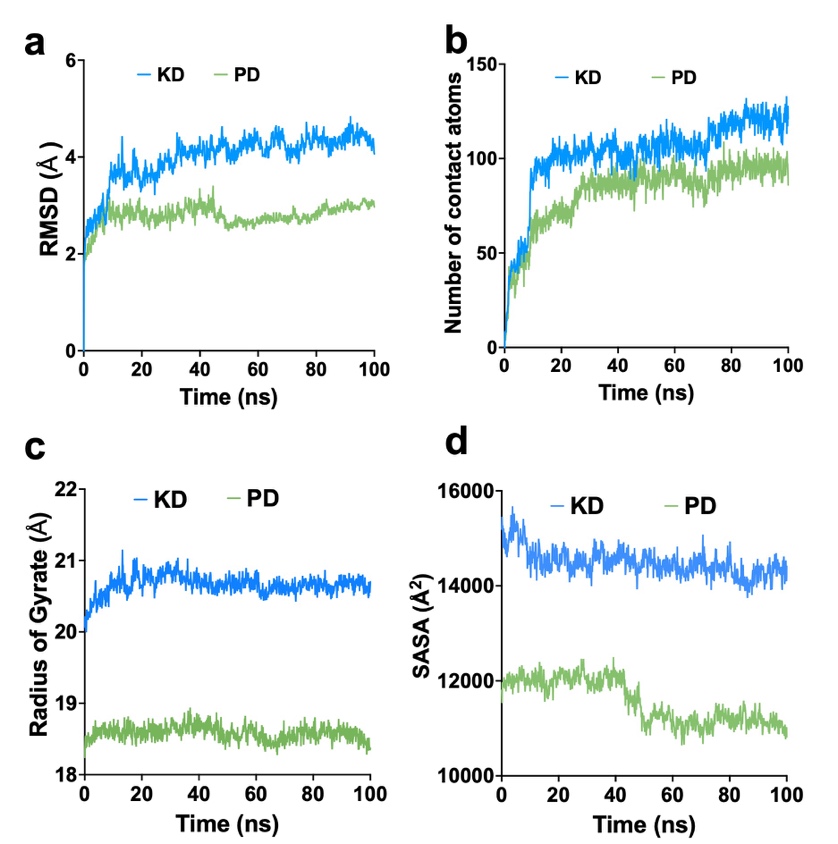


**Figure S6. BPNM binding with PLK1-KD and -PD *in silico.*** a-e) The change of RMSD (b), Number of contact atoms (b), radius of gyrate (c), SASA (d) of PLK1-KD and -PD during 100 ns of MD simulation.

**
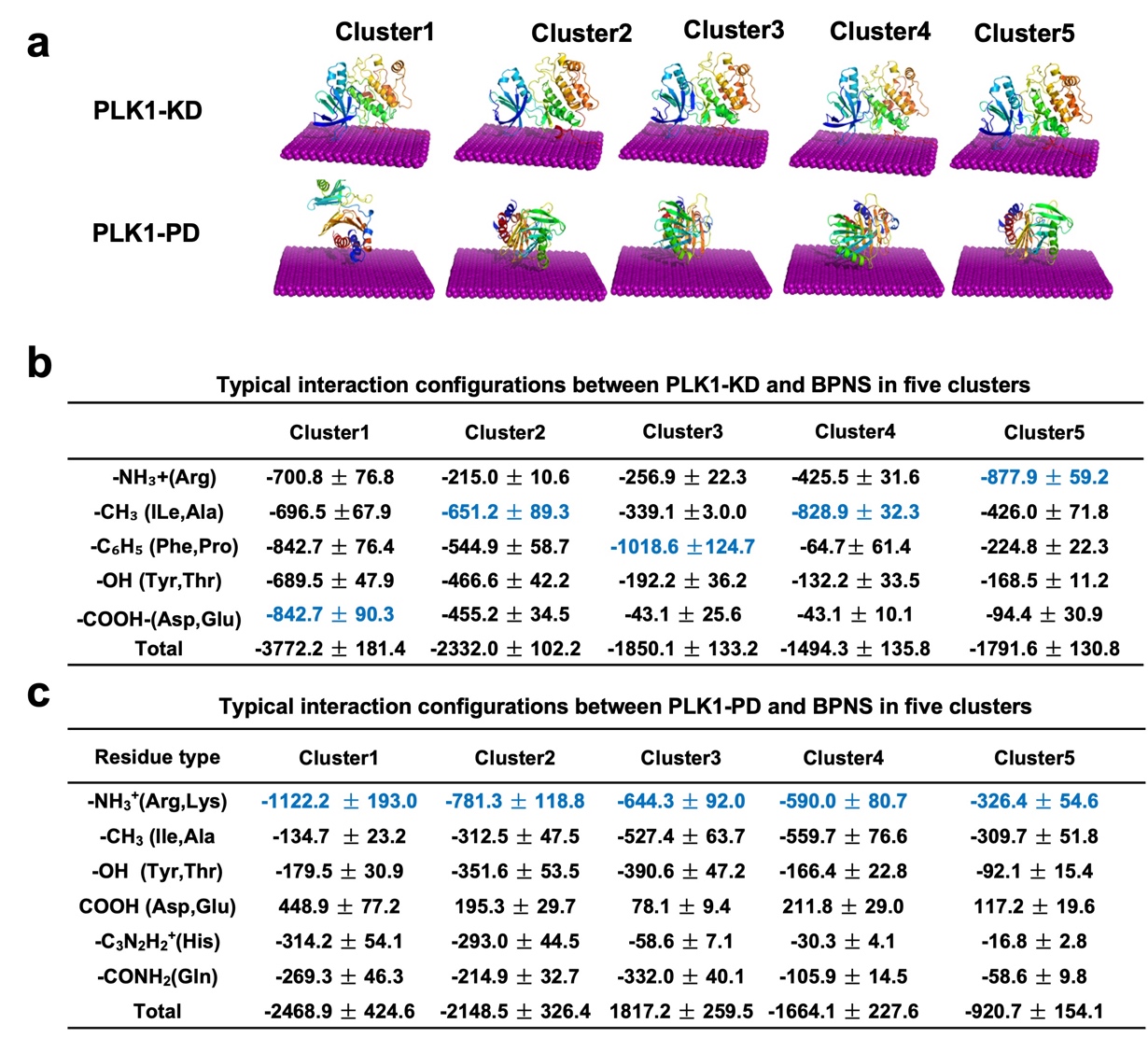
**

**Figure S7. Typical interaction configurations between BPNMs with PLK1-KD and -PD.** a) Five typical interaction configurations (cluster1-5) between BPNMs and PLK1- -KD and -PD. b-d) The interaction energies (KJ/mol) and related amino acid residues between BPNMs and PLK1-KD (b), -PD (c). The highest energy that amino acid residues contribute to a typical interaction configuration is marked in blue.

**
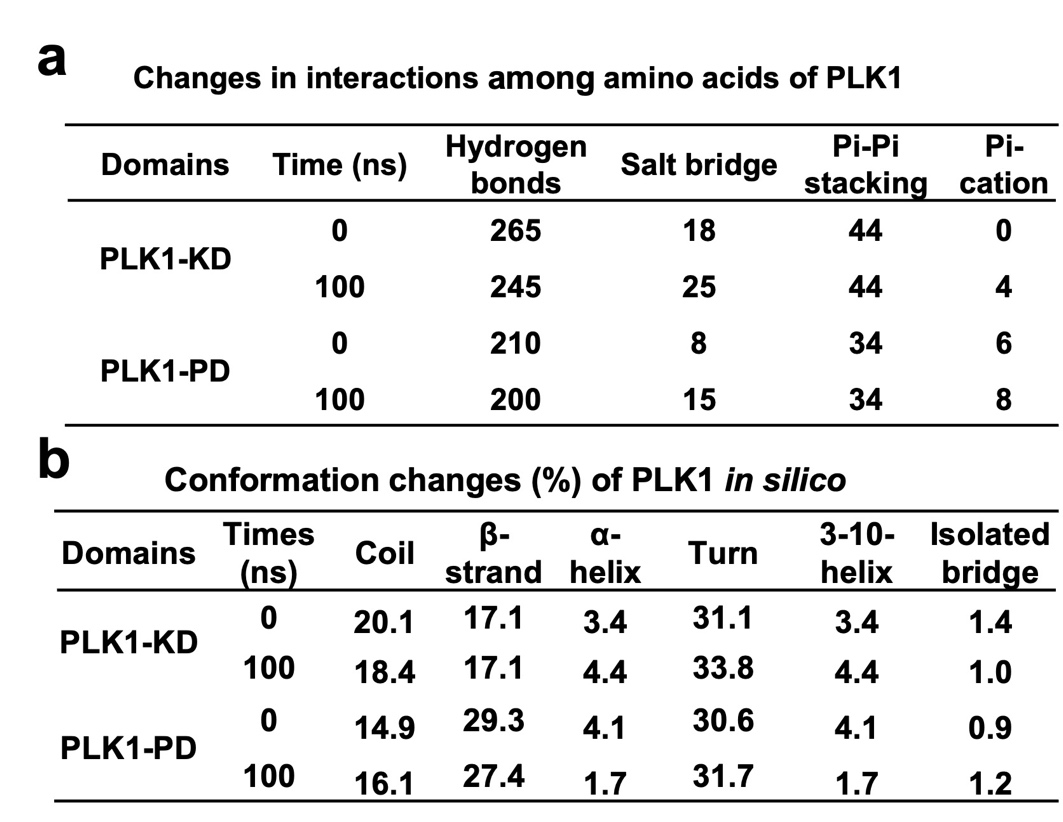
**

**Figure S8. Conformation variation of PLK1-KD and -PD after binding with BPNMs.** a) The change in interactions among amino acids of PLK1-KD and -PD during 100 ns of MD simulation. b) Conformation changes of PLK1 after incubation with BPNMs *in silico*.


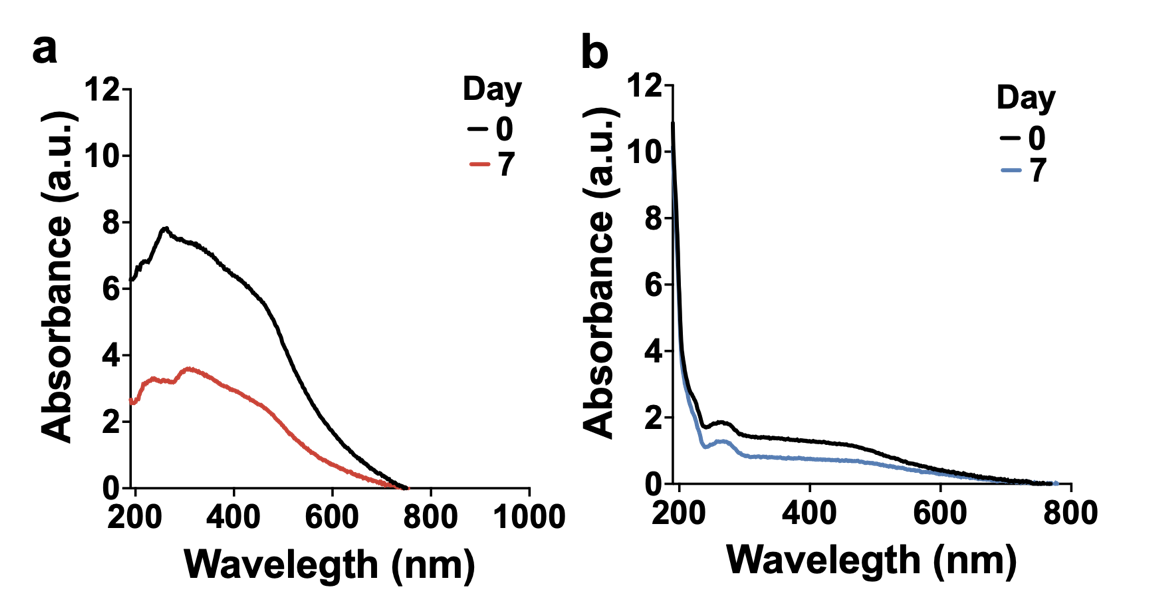


**Fig. S9. The UV-vis absorption spectra of BPQDs (a) and CM-BPQDs (b) in water at day 0 or after 7 days.**

**
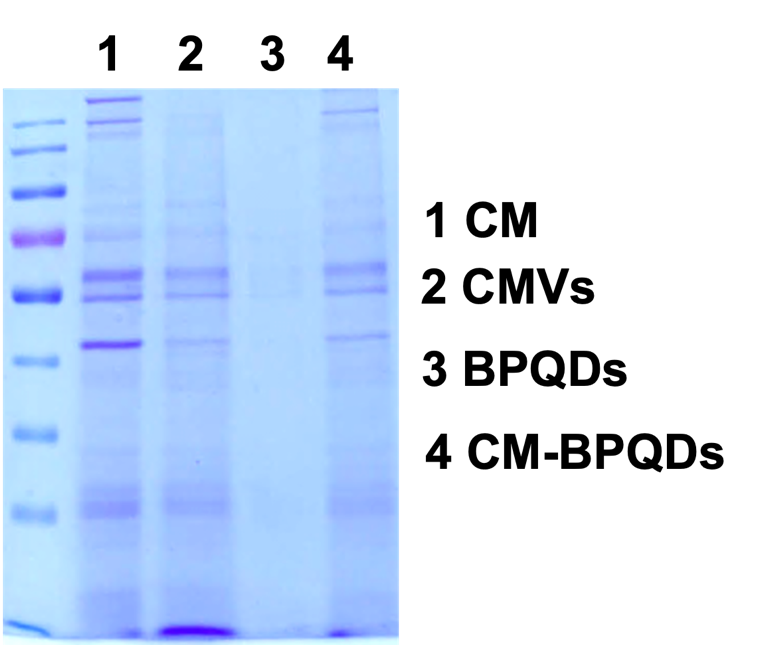
**

**Figure S10. Proteins on cell membranes and CM-BPQDs** (CM, cell membranes extracted from myeloid-derived cells; CMVs, cell membranes vesicles after extrusion；BPQDs; CM-BPQDs).


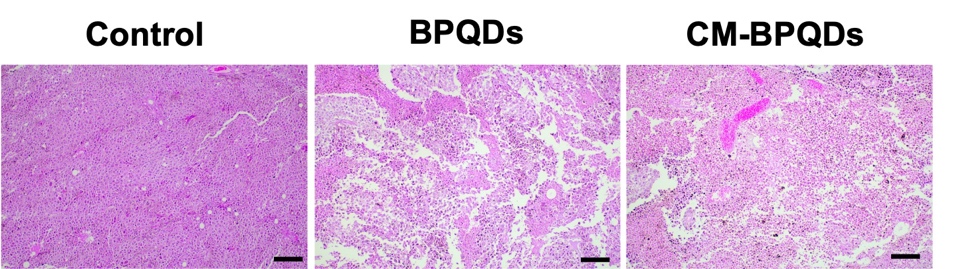


**Figure S11. Images of tumor tissues stained with HE.**

**
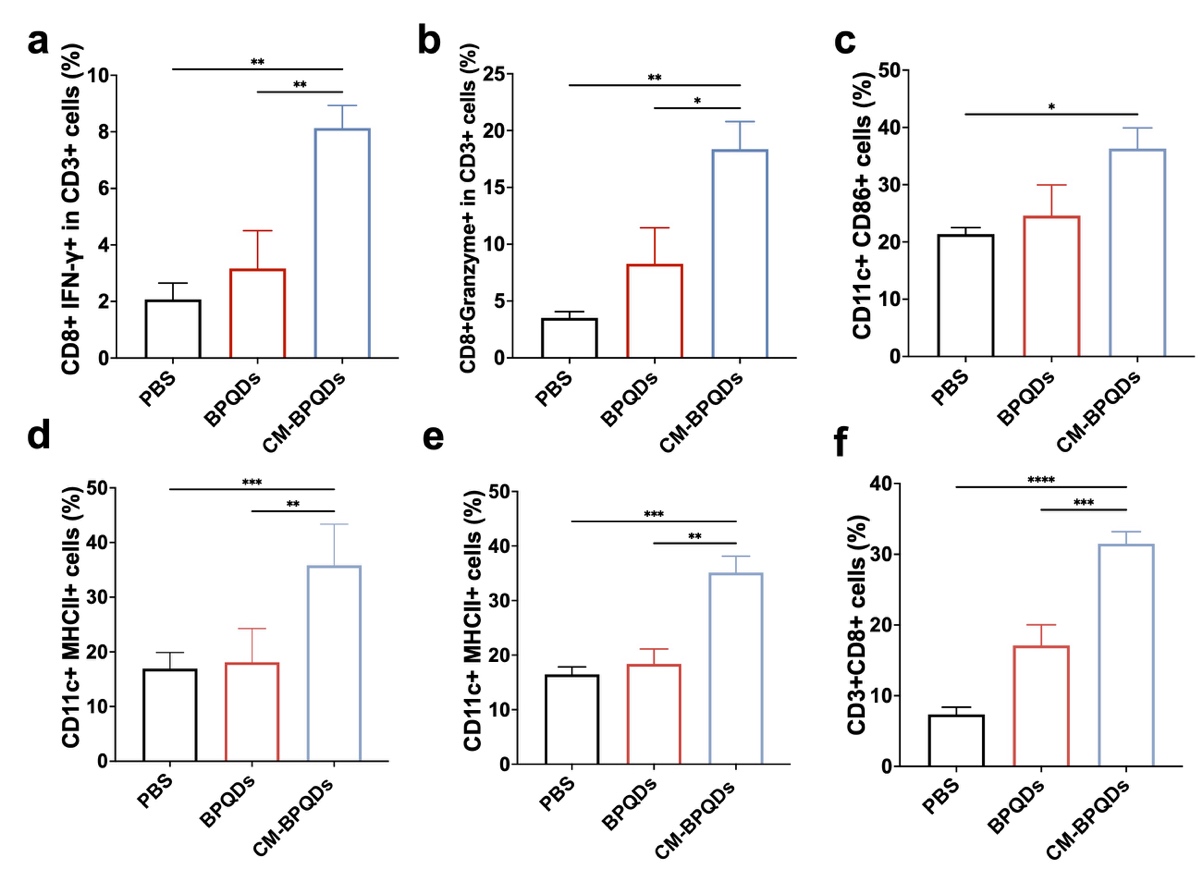
**

**Figure S12. Flow cytometry analysis of immune response in tumor tissues and lymph nodes after systematic administration of CM-BPQDs in melanoma-bearing mice.** a-b) Percentage of CD8+IFN-γ+ and CD8+Granzyme+ in CD3+ cells in tumors after BPQDs and CM-BPQDs treatments in tumor-bearing mice. c-f) Percentages of CD86+ (c), MHCI+ (d), MHCII+ (e) in CD11c+ cells and CD8+ (f) in CD3+ cells in lymph nodes after BPQDs and CM-BPQDs treatments in melanoma-bearing mice. Data are presented as mean ± SEM. **p* < 0.05, ***p*< 0.01, compared with control group.


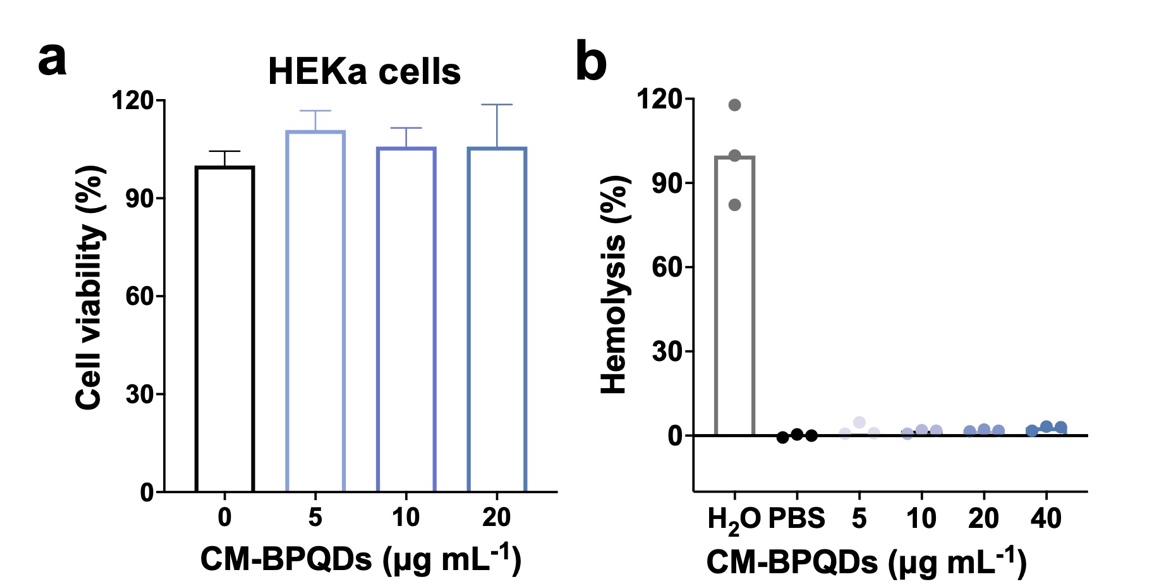


**Figure S13. The safety of CM-BPNMs *in vitro.*** a) The cell viability HEKa cells after incubated with CM-BPQDs for 24 h. b) The percentage of hemolysis of red blood cells after incubated with CM-BPQDs for 2 h at 37℃.
